# Angstrom-Scale Confinement Activates O_2_ via Spin and Charge Reconfiguration

**DOI:** 10.1021/acs.jpclett.6c01844

**Published:** 2026-08-18

**Authors:** Chenjiao Bu, Liangliang Huang, Jianguo Wang

**Affiliations:** † Zhejiang Key Laboratory of Surface and Interface Science and Engineering for Catalysts, College of Chemical Engineering, 12624 Zhejiang University of Technology, Hangzhou 310014, China; ‡ School of Sustainable Chemical, Biological & Materials Engineering, 6187 University of Oklahoma, Norman, Oklahoma 73019, United States

## Abstract

First-principles calculations show that angstrom-scale slit confinement alone activates O_2_ via the coupled spin, charge, and bonding reconfiguration. Across electronically distinct walls (graphene, h-BN, and g-C_3_N_4_), reducing the gap drives a stepwise evolution from triplet O_2_ to superoxide-like and spin-quenched peroxo-like states, followed by O–O scission and surface chemisorption. A pressure-gap relation exhibits a sharp signature at the crossover, suggesting the confinement-driven generation of reactive oxygen species without dopants, photons, or external fields.

## Graphical abstract

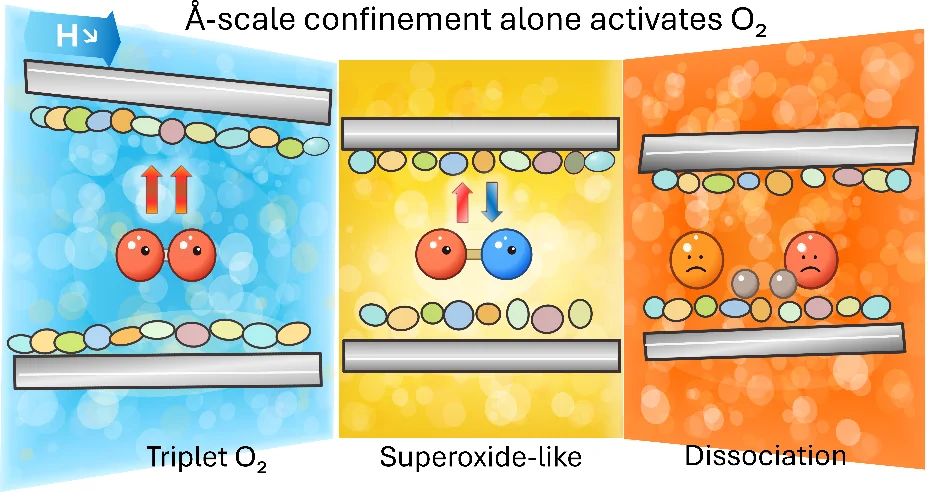

Chemical reactions underpin the transformation of matter and energy across nature, technology, and living systems.^1^ Electron-transfer processes are especially influential because they regulate electronic occupations and transport in chemical and electrochemical environments.^2,3^ Culminating in Marcus theory,^4^ thermodynamic driving forces and kinetic rate laws for electron transfer have been broadly formulated and validated.^5,6^ Molecular oxygen is particularly exceptional because its redox chemistry is intrinsically coupled to its spin state. O_2_ spans multiple spin manifolds and accessible oxidation states, enabling roles that range from terminal oxidant to diverse reactive oxygen species (ROS).^7,8^ Spin-selection rules, competition between one-electron and two-electron (often proton-coupled)^4,9,10^ pathways, and transient charge-transfer configurations make O_2_ chemistry highly sensitive to how frontier states are split and mixed. In the linear-combination-of-atomic-orbitals (LCAO) framework,^11^ such mixing directly reshapes bonding and antibonding character through orbital-phase interference,^12^ suggesting that geometry alone can, in principle, reorganize spin, charge, and bonding.

Conventional routes that convert stable triplet O_2_ to ROS typically rely on catalysts or external stimuli, such as dopants/defects,^13–15^ photoexcitation,^16–19^ or plasmas.^20,21^ Related first-principles studies of graphene-based adsorption and O_2_ activation have shown that adsorption geometry, frontier-orbital alignment, heteroatom doping, topological defects, and metal–graphene hybridization can strongly affect O_2_ binding, O–O elongation, and dissociative chemisorption.^22–27^ These approaches mix spin states and promote charge transfer, helping to overcome spin-selection constraints.^28,29^ This motivates a fundamental question: to what extent can angstrom-scale geometric confinement alone, independent of specific host chemistry, reconfigure the spin and orbital occupations of O_2_ to promote activation? To probe this while separating confinement physics from chemical complexity, we adopt a model van der Waals slit formed by two rigid, pristine 2D sheets separated by a tunable gap *H* (Figure S1). To test generality, we use three electronically distinct but defect-free walls, namely, graphene, hexagonal boron nitride, and graphitic C_3_N_4_, while holding the walls rigid so that *H* is the sole control parameter. A single O_2_ molecule is placed within the slit, and its atomic coordinates are relaxed independently at each fixed H, providing a quasi-static map of its spin, charge, and bonding evolution under confinement; pore-entry dynamics, O–O dissociation kinetics, and wall reconstruction are beyond the scope of the present rigid-wall model and are reserved for future study. This design connects to mechanochemical activation in a quasi-2D setting,^30–32^ where molecules trapped between atomically thin layers can experience effective pressures of several gigapascals.^33–35^

This Letter reports that angstrom-scale slit confinement alone drives a stepwise reconfiguration of the spin state, charge distribution, and bonding of O_2_ across graphene, h-BN, and g-C_3_N_4_. As *H* decreases, O_2_ evolves from a stable triplet to a superoxide-like intermediate, then to a spin-quenched peroxo-like state, and finally to O–O scission with wall–O chemisorption. We quantify this progression using four primary descriptors: the total spin magnetic moment (*M*_*S*_), Bader charge transfer (Δ*q*),^36,37^ O–O bond length (*l*_O–O_), and bonding metrics from the crystal orbital Hamilton population (COHP)/crystal orbital bond index (COBI) and their energy integrals (ICOHP/ICOBI).^12,38–41^ A molecule-fixed σ/π orbital analysis can link the transitions to confinement-induced splitting and progressive occupation of π* antibonding levels that weaken and ultimately cleave the O–O bond. Finally, the excess normal pressure *P*_*zz*_^(ex)^(*H*), obtained from the adsorption-energy slope with respect to *H*, provides a mechanical correlate of the electronic crossover and suggests experimentally accessible signatures. Together, these elements establish field-free geometric confinement as a general control parameter for ROS-relevant activation of molecular oxygen.

## Confinement-Controlled State Map of O_2_

In isolation, molecular oxygen is a triplet with two unpaired electrons in degenerate π* orbitals.^42^ We quantify how angstrom-scale slit confinement modifies this electronic structure by scanning the wall gap *H*. Figure 1 summarizes the evolution of the total spin moment *M*_*S*_, Bader charge transfer Δ*q*, and O–O bond length *l*_O–O_ for the graphene slit. The same sequence of states is obtained for h-BN and g-C_3_N_4_; more details are available from Figure S1 and Table 1.

**Figure 1:**
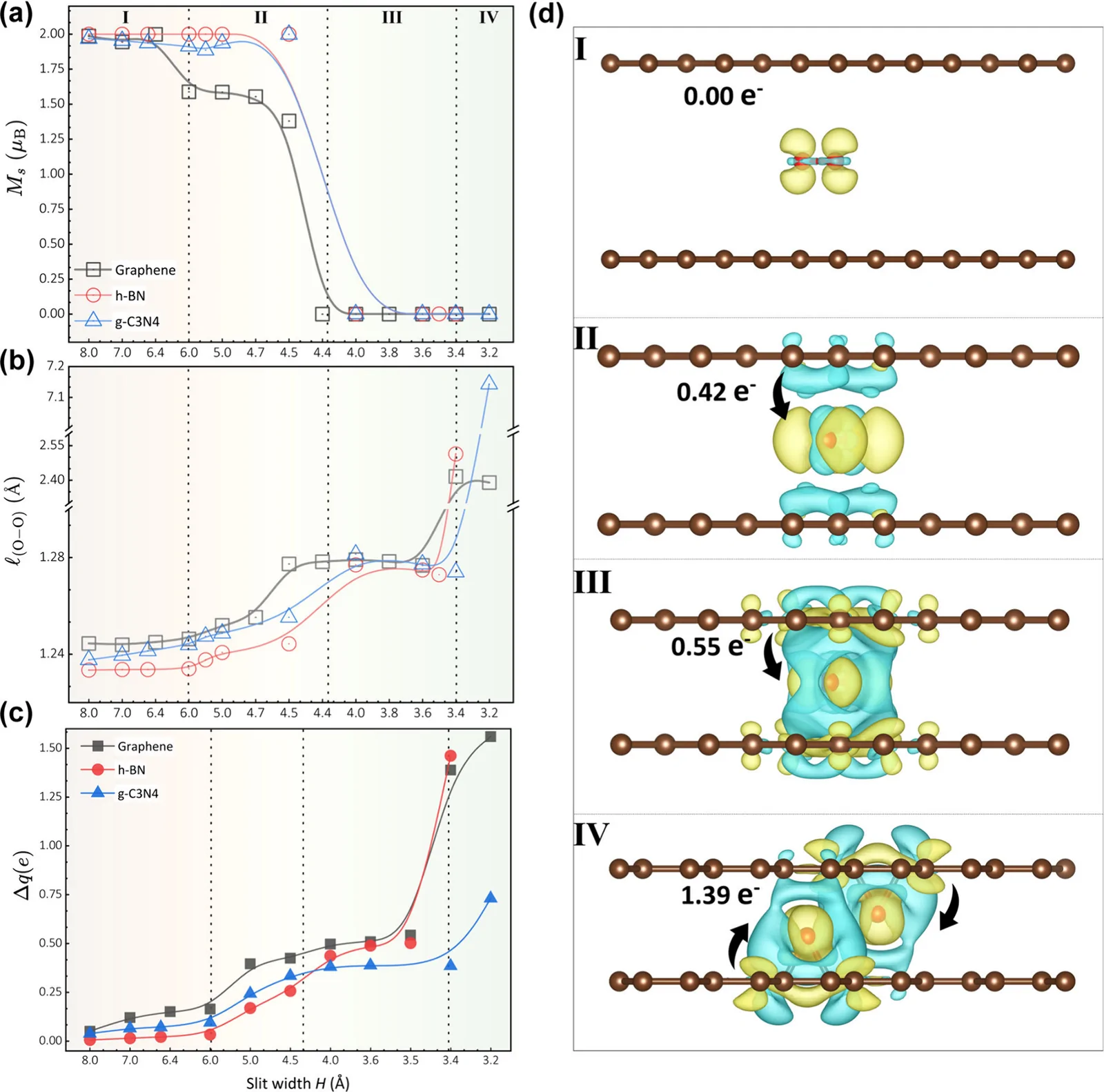
Confinement-controlled state map of O_2_ vs slit width *H*. (a) *M*_*S*_(μ_*B*_), (b) *l*_O–O_ (Å), and (c) Δ*q*. (d) Charge-redistribution isosurfaces Δρ (O_2_ confined in the graphene slit pore; accumulation, yellow; depletion, cyan; see Figure S2 for h-BN and g-C_3_N_4_).^43^ The shading marks regimes I–IV (triplet → superoxide-like → spin-quenched/peroxo-like → dissociated). Points report the lowest-energy state at each *H*. Previous studies have reported tilted O_2_ adsorption geometries on graphene surfaces.^44^ In the present three-walled system, sampling multiple initial orientations and lateral positions yielded lowest-energy structures with final angles of 0.00, 0.04, and 0.00° at *H* = 3.4, 4.5, and 6.0 Å, respectively, indicating a relaxation-driven preference for a nearly parallel orientation. The confining walls are rigid and electronically passive; *H* is the sole control parameter.

**Table 1:** Confinement Thresholds for O_2_ Activation across Host Materials^a^

Host	*H* at *M*_*S*_ → 0 (Å)	H at O–O cleavage (Å)
Graphene	4.4	3.4
h-BN	4.0	3.4
g-C_3_N_4_	4.0	3.2

a Approximate slit gaps *H* at which (i) the total moment collapses (*M*_*S*_ → 0, from regime II to III) and (ii) O–O bond cleavage is observed from regime III to IV. Detailed regime-resolved descriptor ranges are provided in Figure 1, Figures S2–S6, and Table S5.

At large gaps (*H* ≳ 7–8 Å), O_2_ remains a weakly perturbed triplet. The spin moment stays near 2 μ_*B*_, and charge transfer is negligible. The computed O–O bond length is close to 1.24 Å, consistent with prior DFT and experimental gas-phase values.^45–47^ As *H* decreases into the intermediate range (∼6.0–4.5 Å), confinement stabilizes partial electron transfer from the walls into O_2_. One π* orbital becomes preferentially occupied, and one spin is paired. *M*_*S*_ decreases, and the O–O bond elongates.^48^ These signatures are consistent with a superoxide-like O_2_^–^ intermediate.^49–51^ Upon further compression, the system undergoes a second reduction step. Near the crossover gap (*H* ≈ 4.4 Å in graphene and ∼4.0 Å in h-BN and g-C_3_N_4_), the total moment collapses to zero. Additional charge transfer fills the second π* orbital and pairs the remaining electron. The resulting closed-shell configuration is a charge-transfer-stabilized, peroxo-like O_2_^2–^ state.^52^ Additional noncollinear SOC calculations preserve this confinement-dependent spin-state evolution: the structures at *H* = 4.0 Å remain spin-quenched, whereas those at *H* = 4.5 and 6.0 Å retain triplet-like magnetizations, with transverse components below 1.0 × 10^–3^μ_*B*_ (Table S6). Evolution of the spin-density distribution, Δρ_*s*_(**r**) = ρ_↑_(**r**) – ρ_↓_(**r**), for O_2_ confined between graphene walls at *H* = 7.0, 4.5, and 3.6 Å is shown in Figure S8. The adsorption-energy profile steepens near this crossover (Figure S3), consistent with the energetic cost of spin pairing and bond reorganization.^53^ At the smallest gaps, O–O cleavage becomes favorable. Further reduction of *H* drives strong O–wall interactions and the O–O bond cleavage. The oxygen atoms migrate toward the walls and chemisorb, yielding dissociated surface oxygen (case 2 in Figure S1).^54^ We therefore identify four distinguishable confinement regimes, as illustrated in Figure 1: (I) triplet O_2_, (II) superoxide-like O_2_^–^, (III) spin-quenched peroxo-like O_2_^2–^, and (IV) dissociated chemisorption.

## Orbital Mechanism: Confinement-Driven π* Filling and σ Collapse

To expose the microscopic origin of the state sequence in Figure 1, we analyze the O–O interaction in a molecule-fixed frame so that “along-bond” (σ) and “transverse” (π/π*) retain their meaning even as O_2_ tilts. Kohn–Sham states are projected onto O-centered Löwdin orbitals using the LOBSTER code.^48,55^ The O 2p basis is rotated such that the *z* ^′^ axis coincides with the instantaneous O–O bond. We then group the contributions into three symmetry channels (Figure 2a): σ­(2s), σ­(p–p) from axial 2p_*z* ^′^_–2p_*z* ^′^_, and π/π* from transverse 2p_*x* ^′^_, 2p_*y* ^′^_.

**Figure 2:**
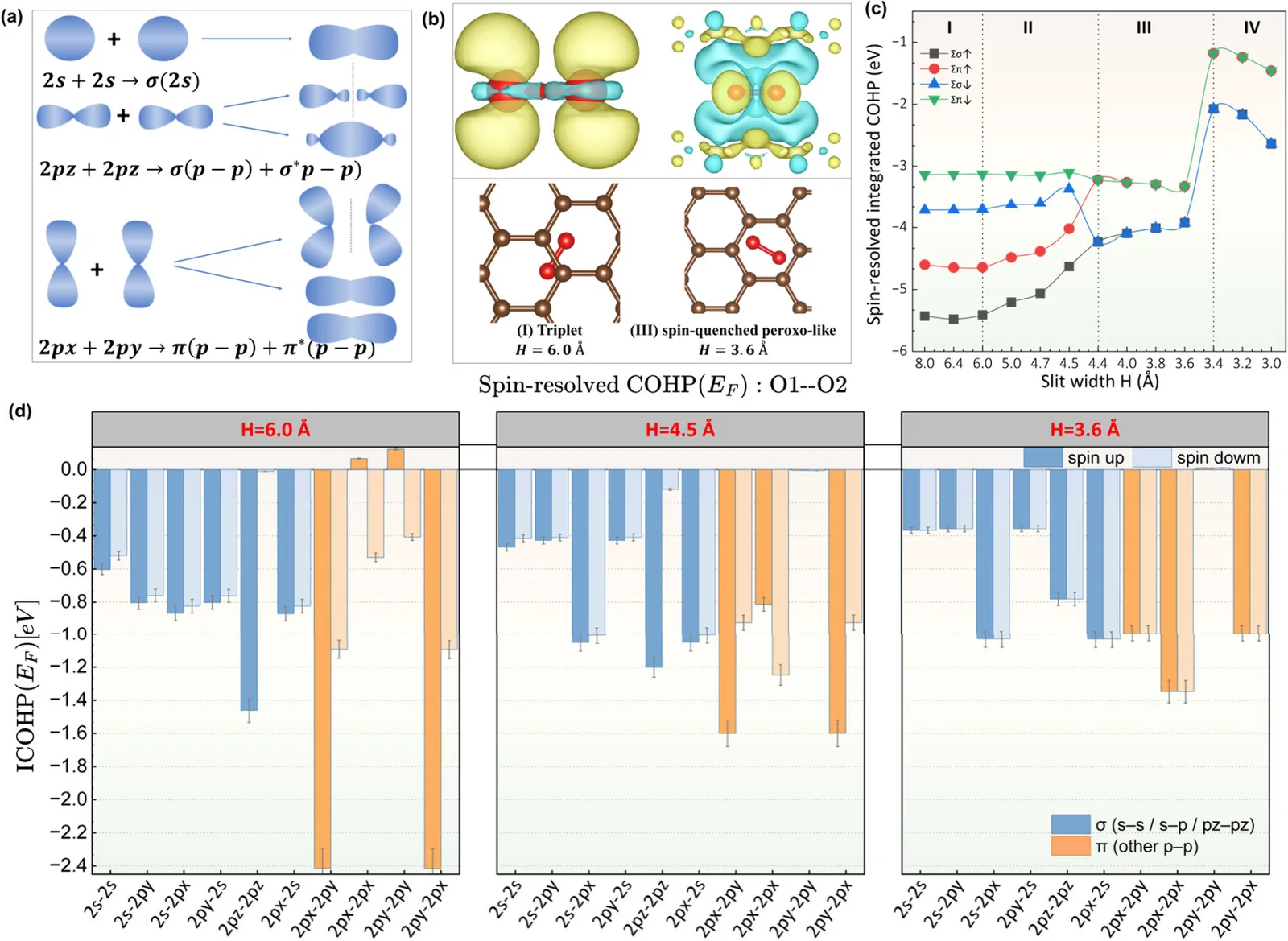
Orbital- and spin-resolved mechanism of confinement-driven O_2_ activation. (a) Molecule-fixed orbital frame and σ/π orbital definitions used for the projection. (b) Spin density illustrating the evolution from triplet to spin-quenched states. (c) Spin-resolved and total ICOHP versus *H* for the O–O bond. The less negative values indicate bond weakening. After O–O cleavage, a strong wall–O ICOHP appears. (d) Orbital-pair-resolved COHP at representative gaps for regimes I–III, showing progressive π* antibonding occupation and the loss of σ bonding.

At large gaps in regime I, the electronic structure remains essentially that of gas-phase triplet O_2_. The spin density is symmetric on the two O atoms (Figure 2b). The bonding analysis shows that the O–O interaction is dominated by the axial σ­(*p*–*p*)­channel, while the two π* orbitals remain half-occupied with parallel spins (Figure 2c,d). This is the baseline configuration from which confinement drives activation. As the slit narrows into regime II, confinement lifts the π* degeneracy and stabilizes charge transfer into one π* orbital. This preferential π* occupation pairs one of the two unpaired electrons and reduces *M*_*S*_. In the bonding decomposition, the key signature is the progressive loss of spin asymmetry in the π-resolved ICOHP together with a weakening of the net O–O bonding (Figure 2c). Importantly, the evolution is continuous with *H*. The bond destabilizes through gradual π* filling and rebalancing between σ and π/π* orbitals, not through an abrupt geometric distortion.

In regime III, additional charge transfer fills the second π* orbital and removes the remaining spin polarization. The resulting spin-quenched configuration is a charge-transfer-stabilized peroxo-like O_2_ state, and it coincides with a pronounced reduction of the axial σ­(p–p) bonding contribution. The O–O ICOHP becomes significantly less negative (Figure 2c), consistent with the collapse of covalent bond order as antibonding π* weight accumulates near and below the Fermi level (Figure 2d). In regime IV, O–O bond cleavage occurs and the intramolecular σ/π decomposition is no longer meaningful. Bonding instead switches to strong wall–O interactions. This is captured by the emergence of a large negative wall–O ICOHP and new bonding features in the energy-resolved COHP after decomposition (Figure 2c, d). Thus, the confinement-dependent state activation is truly governed by a single orbital-level mechanism: confinement stabilizes π* antibonding occupation, which weakens the axial σ bond, quenches the spin upon π* pairing, and ultimately enables wall–O bond formation after O–O scission. The same sequence of π*-driven orbital reorganization and O–O bond weakening is obtained for h-BN and g-C_3_N_4_ (Figures S4 and S5), with only modest shifts in the critical gaps. This confirms that the mechanism is set primarily by angstrom-scale geometry rather than by a specific wall chemistry.

Figure 3 provides energy-resolved bonding evidence for the confinement-driven state sequence in Figure 1. In regime I, the O–O π* antibonding contribution lies above *E*_*F*_ with small weight, consistent with a stable molecular bond and a large O–O bond index. In regime II, the π* feature shifts downward and gains intensity near *E*_*F*_. This indicates partial occupation of antibonding states and coincides with the superoxide-like regime. In regime III, π* weight moves to and across *E*_*F*_, so antibonding occupation becomes substantial. Meanwhile, the COBI simultaneously drops further, consistent with strong O–O weakening in the spin-quenched, peroxo-like state. Eventually, the O–O bonding is lost and new wall–O bonding features appear in regime IV, marking the transition to dissociative chemisorption. Equivalent COBI trends for h-BN and g-C_3_N_4_ confirm that this π*-driven weakening mechanism has been observed for all three materials (Figures S6 and S7). It is also worth noting that the confinement crossover involves an energy scale of order 1 eV, consistent with the cost of spin pairing in O_2_ and the known triplet–singlet energetic separation.^56,57^

**Figure 3:**
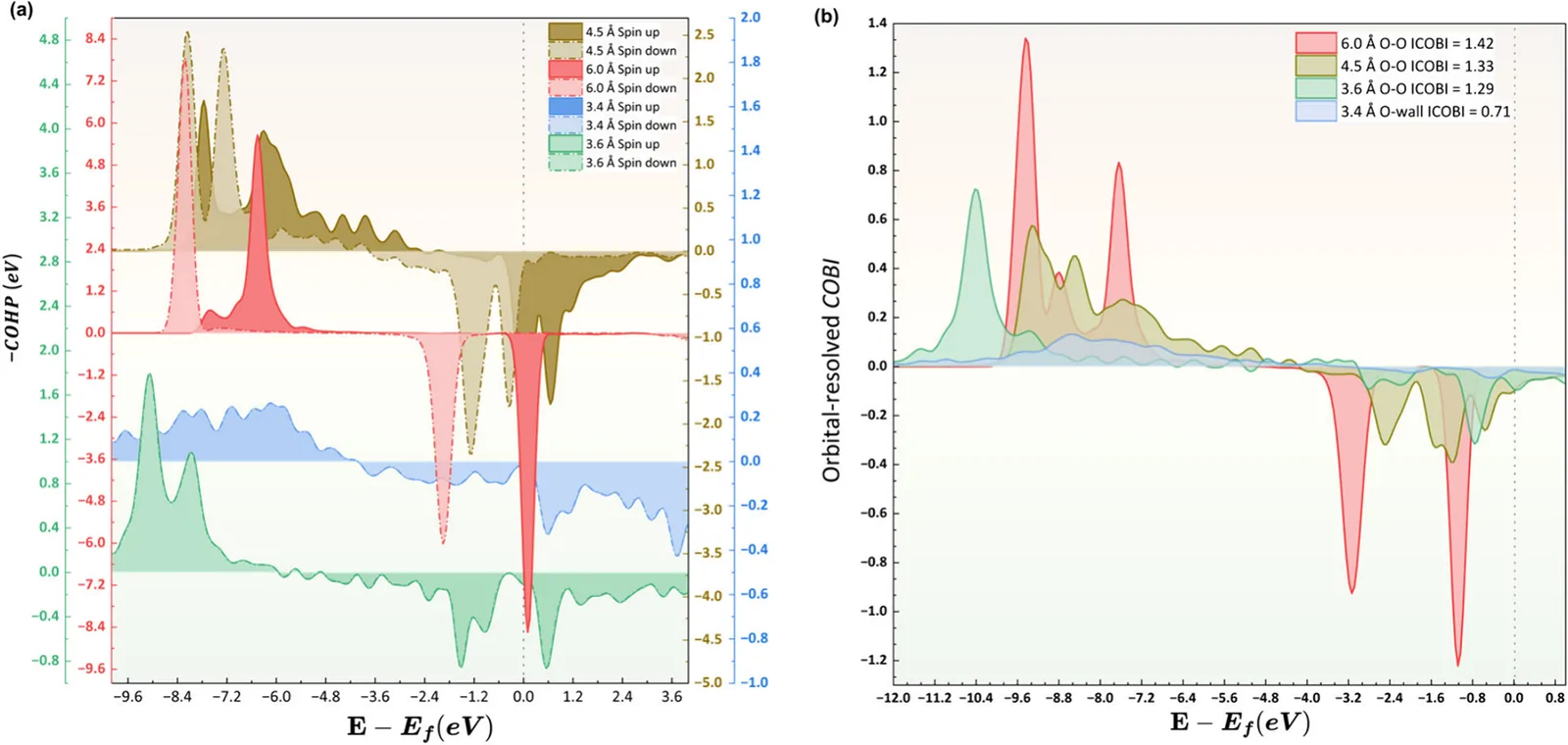
COHP/COBI signatures of confinement-driven O–O weakening and wall–O bond formation (graphene slit). (a, b) Regimes I–IV, respectively. Note that −COHP­(E) is plotted so that positive values indicate bonding and negative values indicate antibonding contributions. With decreasing gap *H*, the O–O π* antibonding feature shifts toward *E*_*F*_ and gains weight, indicating increasing antibonding occupation and progressive O–O bond weakening. The corresponding energy-resolved COBI shows a monotonic reduction of the O–O bond index across regimes I–III (ICOBI decreases from ∼1.5 to ∼1.3). After O–O cleavage (regime IV), the O–O ICOBI vanishes while a finite wall–O bond index emerges. COHP and COBI trends for h-BN and g-C_3_N_4_ are shown in Figures S5 and S6.

## Mechanical Signature: Excess Normal Pressure under Confinement

To map the geometric control parameter *H* onto a mechanical load, we evaluate the excess normal pressure as

$$P_{zz}^{\left(ex\right)}\left(H\right)=-\frac{1}{A}\frac{dE_{ads}\left(H\right)}{dH}$$

where *E*_ads_(*H*) is the adsorption energy at fixed *H* and *A* is the in-plane cell area.^58,59^ Figure 4 shows *P*_*zz*_^(ex)^(*H*) for the three materials. The normal pressure is small in the triplet regime (regime I) and then rises rapidly as the gap narrows. A step-like increase occurs at the spin/charge crossover from regime II to III, providing a mechanical marker of the electronic transition. Upon further compression, *P*_*zz*_^(ex)^ reaches a maximum in the spin-quenched peroxo-like regime just before O–O bond cleavage and then decreases after cleavage as the stress is accommodated by localized wall–O bonding. Over regimes I–III, *P*_*zz*_^(ex)^ is well described by a single-exponential dependence on *H*,^60^ consistent with short-range overlap repulsion. The same signature also appears for h-BN and g-C_3_N_4_: small load at large *H*, a crossover jump, and a precleavage maximum, indicating that the mechanical correlation is robust across host materials.^61^

**Figure 4:**
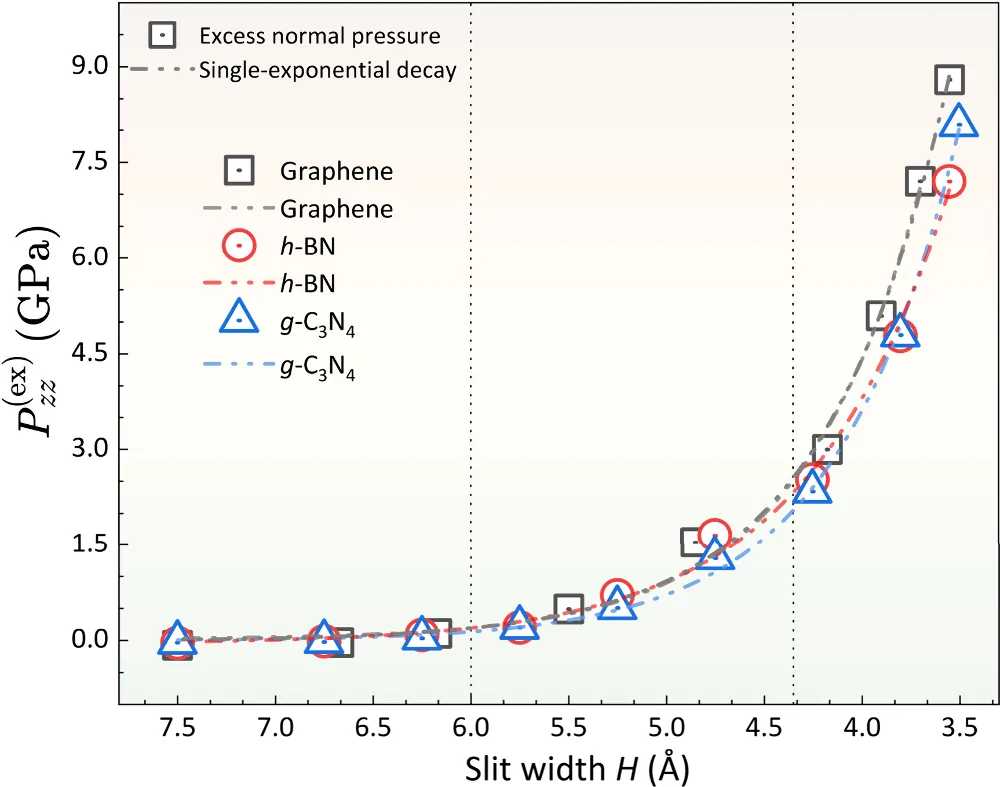
Excess normal pressure *P*_*zz*_^(ex)^(*H*) extracted from the adsorption-energy slopes. The horizontal axis is inverted so that increasing compression corresponds to decreasing *H*. The load is small in the triplet regime, showing a step-like increase at the spin/charge crossover (*H* ≈ 4–4.5 Å) and peaking in the peroxo-like regime just before O–O bond cleavage (*H* ≈ 3.6 Å).

## Materials Independent of Confinement-Induced O_2_ Activation

Graphene, h-BN, and g-C_3_N_4_ span distinct electronic structures^62^ (see Figure S7 and Table S4). Nevertheless, all three hosts exhibit the same confinement-driven sequence of O_2_ states identified in Figure 1. Table 1 summarizes two characteristic gap thresholds for each host: the spin/charge crossover into the spin-quenched, peroxo-like regime (regime II to III) and the onset of O–O bond cleavage (regime III to IV). The differences are modest for the two shifts. Graphene shows earlier charge uptake at a given *H*, whereas h-BN and g-C_3_N_4_ shift the crossover to slightly smaller gaps (Figure 1), consistent with differences in electron donation and mechanical compliance of the normal pressure. The persistence of the same regime order across these electronically distinct walls supports a geometry-governed field-free activation pathway.

Approximate spin projection in periodic plane-wave DFT assumes that a broken-symmetry solution is dominated by a two-state mixture of the target low-spin state and a corresponding high-spin reference, thereby neglecting possible contributions from other spin states or multiconfigurational character. For confined O_2_, however, this mapping becomes ambiguous as charge transfer, spin polarization, and O–O bonding evolve with the slit width, *H*. Consequently, no single physically consistent high-spin reference applies throughout the molecular-to-dissociated sequence. Thus, the terms “triplet-like,” “partially spin-polarized,” and “spin-quenched” describe the calculated magnetic-moment and spin-density patterns rather than rigorous spin-pure eigenstates, while residual spin mixing may affect quantitative energies and the precise crossover threshold.^63,64^ Additional constrained HSE06-D3­(BJ) calculations yield a magnetic molecular state at *H* = 4.5 Å and a spin-quenched state at *H* = 4.0 Å for all three wall materials, bracketing the crossover within 4.0 < *H*_c_ < 4.5 Å.^65–67^ These results support the qualitative robustness of the PBE-D3­(BJ) state sequence while indicating that the precise crossover threshold remains quantitatively dependent on the exchange–correlation functional and computational conditions (Table S8).

This Letter shows that angstrom-scale slit confinement, with the gap *H* as the sole control parameter, can activate a single O_2_ molecule under field-free conditions by reconfiguring its spin, charge, and bonding. Across graphene, h-BN, and g-C_3_N_4_, decreasing *H* drives the same sequence of states: triplet physisorption, a superoxide-like intermediate, a spin-quenched peroxo-like charge-transfer state, and finally O–O bond cleavage with wall–O chemisorption. Bonding descriptors, such as COHP/COBI and their integrals, track a monotonic weakening of the O–O bond across the molecular regimes and the emergence of strong wall–O bonding after cleavage. A molecule-fixed σ/π analysis identifies the microscopic mechanism in which confinement stabilizes progressive occupation and pairing in π* antibonding orbitals, suppressing the axial σ bond and quenching the spin. The same transition is accompanied by a mechanical signature in the excess normal pressure *P*_*zz*_^(ex)^(*H*), which is small at large *H*, shows a step-like increase at the crossover, and peaks immediately before O–O cleavage. In realistic materials, the uptake of O_2_ would likely occur through wider edge openings or defects. It could also occur during assembly, followed by compression or local gap reduction, which would drive the confined molecule into the activation regime identified here.

These results suggest practical design rules for van der Waals slits and interlayer materials. Gaps near the crossover (∼4–4.5 Å, with modest host-dependent shifts) stabilize reduced low-spin O_2_ without dissociation, whereas further compression to ∼3.2–3.4 Å promotes O–O cleavage and stable wall–O species. Future work that includes wall flexibility, finite temperature and coverage, and dynamical compression can refine quantitative thresholds and connect the pressure signature to direct experimental observables. The same combined state map, bonding, and pressure framework can be extended to other confined small molecules and slit-pore architectures.

## Supplementary Material





## Notes

Computational details; optimized structures; charge transfer, adsorption energy, COHP/COBI, electronic structure, and spin-density analyses; and supplementary energetic, mechanical, bonding, magnetic moment, spin–orbit coupling, HSE06-D3­(BJ), and orientation/site data (PDF)
Representative computational data and analysis codes for graphene-confined O_2_ at H = 3.6 Å (ZIP)
