# Supplementary material for: Angstrom-Scale Confinement Activates O2 via Spin and Charge Reconfiguration
Source: J Phys Chem Lett. 2026 Aug 18;17(40):11520–6. doi: 10.1021/acs.jpclett.6c01844 (PMC13656783; doi:10.1021/acs.jpclett.6c01844)
Supplement: Supplementary file 1 [file jz6c01844_si_001.pdf]

Supplementary materials:

Å-Scale Confinement Activates O_2 via Spin and Charge Reconfiguration

Chenjiao Bu,[a,b] Liangliang Huang,[b*] Jianguo Wang[a*]

(a) Zhejiang Key Laboratory of Surface and Interface Science and Engineering for Catalysts, College of Chemical Engineering, Zhejiang University of Technology, Hangzhou 310014, China

(b) School of Sustainable Chemical, Biological & Materials Engineering, University of Oklahoma, Norman, OK 73019, USA

1. Summary of computational details

(1) Electronic structure method. Density functional theory (DFT) calculations were performed using the Vienna Ab initio Simulation Package (VASP 6.4.1) within the projector augmented wave (PAW)[1] framework. Exchange–correlation effects were treated using the Perdew–Burke–Ernzerhof (PBE) generalized gradient approximation. Long-range dispersion interactions were included via the Grimme D3 scheme with Becke–Johnson damping (D3(BJ))[2, 3], as implemented in VASP. For geometry optimizations, a plane-wave cutoff of 500 eV were employed. Brillouin-zone sampling used a Γ-centered Monkhorst–Pack grid of 3×3×1 for the C_{120} lateral cell; convergence tests with a 4×4×1 grid altered adsorption energies by less than 5 meV and Bader charges by less than 0.02 e. The electronic convergence and ionic relaxation criteria were established at EDIFF = 1×10^{-5} eV and EDIFFG = –0.02 eV $Å^{-1}$, respectively. The precision setting was designated as PREC = Normal, and the Gaussian smearing scheme with ISMEAR = 0 and SIGMA = 0.01 eV was implemented. The spin-polarization option was enabled ISPIN = 2.

In the context of single-point calculations preceding the LOBSTER projection, a denser electronic accuracy was employed (PREC = Accurate, EDIFF = 1×10^{-6} eV, NELMIN = 4), and symmetry was disabled (ISYM = -1) to ensure compatibility with LOBSTER, as it cannot handle time-reversal symmetry systems. Subsequent chemical bonding analyses were performed using the LOBSTER 5.1.1 code. The pbeVaspFit2015 basis set was utilized, with the inclusion of s and p orbitals in the projection.

(2) Reference energies and adsorption energies. Adsorption energies follow a unified sign convention: $E_{ads}(H) = E_{tot}(H) - E_{walls} - E_{O_2}$ with more negative indicating stronger stabilization by confinement. E_{walls} is the energy of the empty slit (same supercell, same *H*, walls frozen). E_{O_2} is computed in a cubic box of 12×12×20$Å^3$, Γ-only, ENCUT = 500 eV, ISPIN = 2,

with triplet (for ground-state references) or singlet (for diagnostics) multiplicities enforced via initial moments. The lower energy is used in contexts explicitly calling for gas-phase triplet, for example, standard E_{ads} definition relative to triplet O_2.

(3) Charge and magnetization analysis. Bader charge analysis[4] was performed using the Henkelman-group[5] grid-based Bader code (v1.04, 02/08/20) on PAW all-electron densities constructed as $\rho_{ref} = AECCAR0 + AECCAR2$. Charge density is generated using VASPKIT 1.5.1 [6]. The fast Fourier transform (FFT) grids were chosen to ensure well-converged real-space charge densities. For graphene, a grid of 140 × 140 × 224 (NGX × NGY × NGZ) was employed. For h-BN, the FFT grid was set to 96 × 112 × 150, while for g-C_3N_4, a grid of 108 × 108 × 150 was used. To ensure robust basin partitioning, we verified doubling $\frac{NGX}{\frac{Y}{Z}}$changed atomic Bader charges by $< 0.01e$. For spin-polarized runs, initial moments were assigned to O atoms to represent triplet O_2. The total spin moment $M_s(\mu_B)$**,** is taken from OUTCAR. Spin densities are visually inspected to confirm localization on O_2 in molecular regimes. Bader outputs the number of electrons q_i^{Bader} on atom i. For oxygen (PBE PAW valence $Z_{val}^{O} = 6$, the electron uptake on oxygen (unit, e) is:

$$\Delta q_O = \sum_{i \in O} (q_i^{Bader} - Z_{val}^{O} \ )$$

where $\Delta q_O \ > 0$ indicates electron gain by O. Charge transfer for the walls is defined analogously. To obtain a better insight into the charge redistribution after the adsorption processes, we compute the differential charge densities defined as:

$$\Delta\rho \ = \rho_{O_2+wall} - \rho_{wall} \ - \rho_{O_2}$$

where ρ_{O_2+wall} represents the total electronic density for the single-molecule/wall system, ρ_{wall} and ρ_{O_2} are the densities for the isolated walls (in this work it is AA-stacked graphene, AA-stacked h-BN, AA-stacked g-C_3N_4) and the molecule, respectively. The isosurfaces value is 0.0006 $eÅ^{-3}$. Yellow and blue contours represent electron accumulation and depletion, respectively, See Fig. S2.

(4) Chemical Bonding Analysis (LOBSTER[7] Calculations): To analyze the nature of bonding and electronic structure, we employed the LOBSTER v5.1.1 program (Local Orbital Basis Suite)

to project the plane-wave DFT wavefunctions (VASP, PAW-PBE potentials) onto a local atomic orbital basis. The projection basis included C (2s, 2p), B (2s, 2p), N (2s, 2p), and O (2s, 2p) orbitals, depending on the host material (graphene, h-BN, or g-C_3N_4). LOBSTER reconstructs local Hamiltonians via Löwdin orthogonalization, providing access to COHP curves, ICOHP values, and Mulliken-like populations (also known as Gross populations). The COHP method partitions the band-structure energy into contributions from specific atom-atom interactions, providing an energy-resolved view of bonding vs. antibonding states. Following the convention introduced by Dronskowski and coworkers[8]. We define negative COHP values to correspond to bonding interactions (stabilizing), and positive values to antibonding (destabilizing). For clarity of presentation, all COHP plots in this work are shown as –COHP on the horizontal axis, so that bonding contributions appear to the right (positive side) and antibonding to the left. The integrated COHP up to the Fermi level was used as a quantitative measure of bond strength (more negative ICOHP indicates a stronger bond). We computed ICOHP values for the O–O bond within the O_2 molecule as well as for wall–O interactions between oxygen atoms and the nearest substrate atoms (C in graphene, B/N in h-BN, and N in g-C_3N_4). For intact O_2 molecules, $ICOHP_{O-O}$ is reported; after bond rupture, $ICOHP_{wall-O}$ was analyzed instead. Furthermore, LOBSTER's gross population and Mulliken charge analysis were employed to evaluate electron distribution on the O atoms and neighboring substrate atoms, which helps to identify charge transfer and changes in hybridization. Bond identification follows LOBSTER's bond list. To assess the reliability of the orbital projections, we examined the absolute charge-spilling values for representative LOBSTER calculations across different host materials and slit gaps. The absolute charge spilling ranges from 1.04% to 1.61%, with an average value of 1.24%. These consistently low values are well below the commonly used 5% threshold, supporting the reliability of the COHP/COBI and Mulliken population analyses used to describe O–O weakening and wall–O bond formation. Orbital-resolved Mulliken populations were adopted to quantify the molecular σ/π channels in a molecule-fixed frame: the O–O axis defines z, and the O 2p orbitals were rotated accordingly. Contributions were grouped into σ(2s) (2s with σ symmetry), σ(p–p) (2pz–2pz), and π/π^* (2px/2py). All calculations were performed in a spin-polarized framework, with the reported fractions normalized to the total O-centered valence at each confinement distance *H*.

(5) Excess normal pressure from energy slope. The excess normal pressure contributed by the confined molecule is evaluated from the thermodynamic route:

$$P_{zz}^{(ex)}(H) \approx -\frac{1}{A}\frac{dE_{ads}}{dH}$$

Using one-sided branch-aware finite differences on the tabulated $E_{ads}(H)$points (e.g., H= 6.00, 5.00, 4.35, 3.70, 3.60 Å). The conversion $eV\text{Å}^{-3} = 160.22\ GPa$is used. The crossover jumps in $P_{zz}^{(ex)}$reflects the non-smooth slope of$E_{ads}(H)$ at the electronic branch change from triplet to singlet. The post-rupture value at $H \approx 3.4$ Å is not evaluated unless the corresponding E_{ads} point is available. This regime is instead characterized by $ICOPH_{wall-O}$.

(6) Model and boundary conditions. Confinement is modeled as a slit pore formed by two rigid electronically passive aromatic sheets arranged in AA stacking with periodic boundary conditions (see Fig. S1). The optimized lattice constant value for pristine graphene sheets is 2.461Å, corresponding to a C–C bond length of 1.420Å. This value agrees well with experimental values of 2.462Å and 1.421Å[9]. A 20 Å vacuum spacing was applied normally to the wall materials plane to suppress spurious interactions between periodic images. Unless noted, all carbon, boron, and nitrogen atoms were fixed during structural relaxations. A single O_2 molecule was placed between the sheets, and all oxygen coordinates were fully relaxed at each fixed slit width H. In this rigid wall model, all wall atoms are fixed during structural relaxation; therefore, the graphene sheet remains planar by construction. This constraint is used to isolate the effect of H from wall reconstruction, local puckering, and sp^2-to-sp^3 rehybridization, which are known to accompany conventional O_2 chemisorption on activated or metal-supported graphene surfaces[10-12]. In contrast, the O_2 coordinates are fully relaxed at each fixed H; therefore, molecular translation, rotation, tilting, O–O bond elongation, and eventual dissociation remain allowed within the prescribed slit geometry. The lateral supercell contains 60 carbons per sheet (C_{120} total), built from graphene with lattice constant $a = 2.46$ Å. The resulting lateral area is

$$A_{Graphene} = 60 \times \frac{\sqrt{3}}{4}a^2 \approx 157.2\ \text{Å}^2.$$

For h-BN, a hexagonal lattice constant of a = 2.50 Å was used, and a 5 × 5supercell was constructed with

$$A_{h-BN} = 25 \times \frac{\sqrt{3}}{2}a^2 \approx 162.4\ \text{Å}^2.$$

For g-C_3N_4, an AA-stacked layered structure was adopted with lattice constant a = 7.14 Å and a 2×2supercell, resulting in

$$A_{g-C_3N_4} = 4 \times \frac{\sqrt{3}}{2}a^2 \approx 176.7\ \text{Å}^2.$$

The three-dimensional cell is periodic. Along with the confinement direction, the inter-sheet separation H is explicitly set and scanned. We treat H as the sole control parameter. The entry kinetics and the chemical identity of the walls are out of scope by design. All calculations included a dipole correction perpendicular to the layers to avoid spurious interactions due to the asymmetric charge distribution when O_2 is present.

(7) HSE06 validation calculations: To assess the functional sensitivity of the confinement-induced spin transition, additional spin-polarized HSE06-D3(BJ) calculations were performed using VASP[13-15]. The screened hybrid functional was applied with an exact-exchange fraction of 0.25 and a screening parameter of 0.2 Å^{-1}. A plane-wave cutoff energy of 500 eV and Γ-point sampling were employed, and the D3(BJ) dispersion correction was retained consistently with the PBE-D3(BJ) calculations. The HSE06 calculations were initiated from the corresponding PBE-D3(BJ)-optimized structures. The wall atoms and slit width were kept fixed, while only the atomic coordinates of O_2 were relaxed at H = 3.4, 4.0, 4.5, and 6.0 Å. The structural optimizations were continued until the forces on the O atoms were below 0.03 eV Å^{-1}.

2. Supplementary Figures

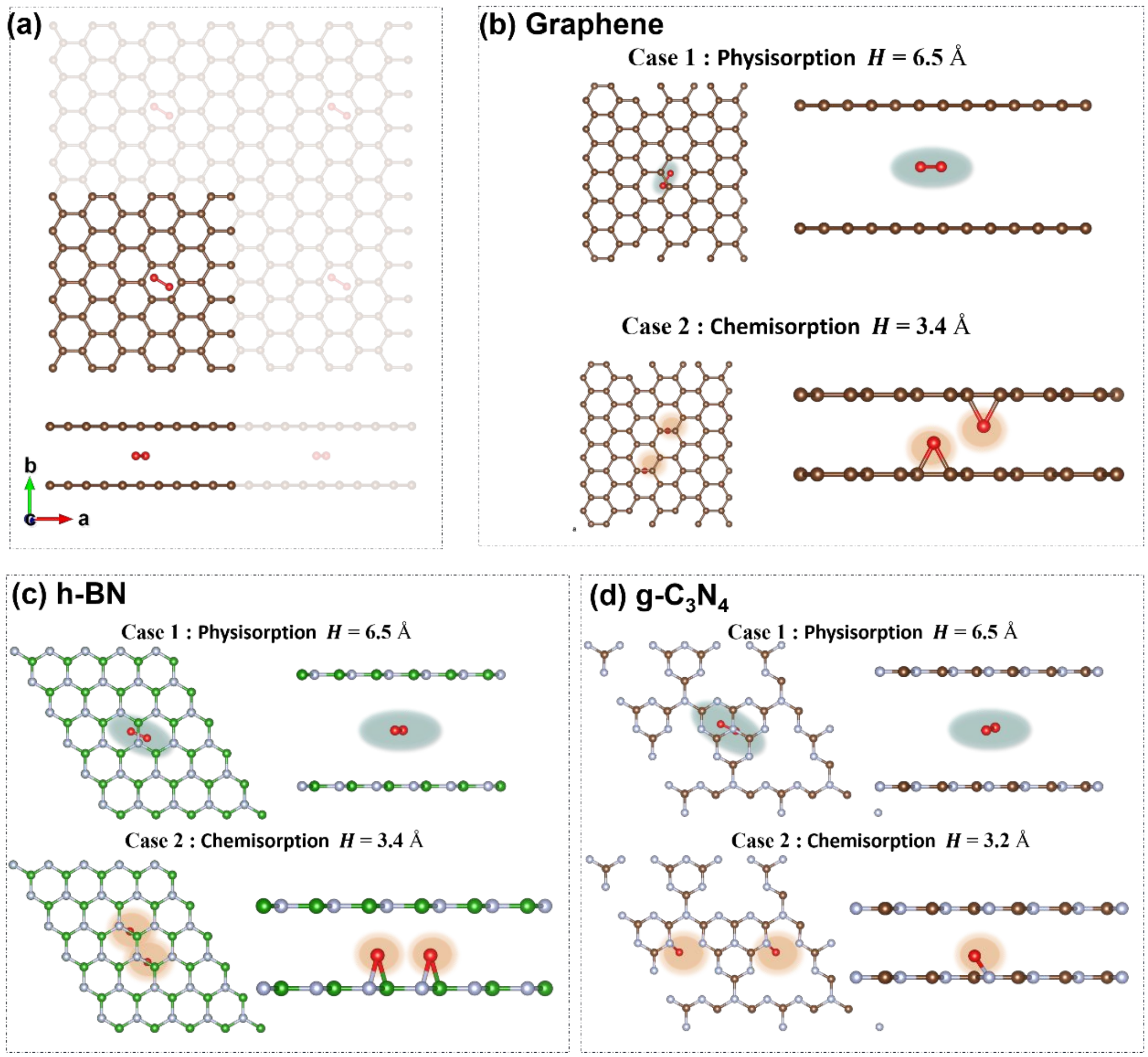

Fig. S1. Optimized model geometries for O_2 confined between bilayers. (a) Supercell setup showing top and side views of the AA-stacked slit system under periodic boundary conditions (PBC). (b–d) Optimized geometries for graphene, h-BN, and g-C_3N_4 walls, each shown at two representative gaps: Case 1, physisorption at large separation ($H = 6.5$ Å); Case 2, chemisorption at compressed separation ($H = 3.4$ Å). Color scheme: C = brown, N = blue, B = green, O = red.

Further compression ($H = 3.4$ / 3.2 Å) leads to each O_2 dissociates and chemisorbs onto the confining walls. In graphene and h-BN, the O atoms bind at bridge-like sites forming epoxide motifs, consistent with prior theoretical reports for O_2 decomposition[16]; in contrast, on g-C_3N_4 material, O atoms anchor to specific reactive edge sites (N), reflecting the localized chemical environment.

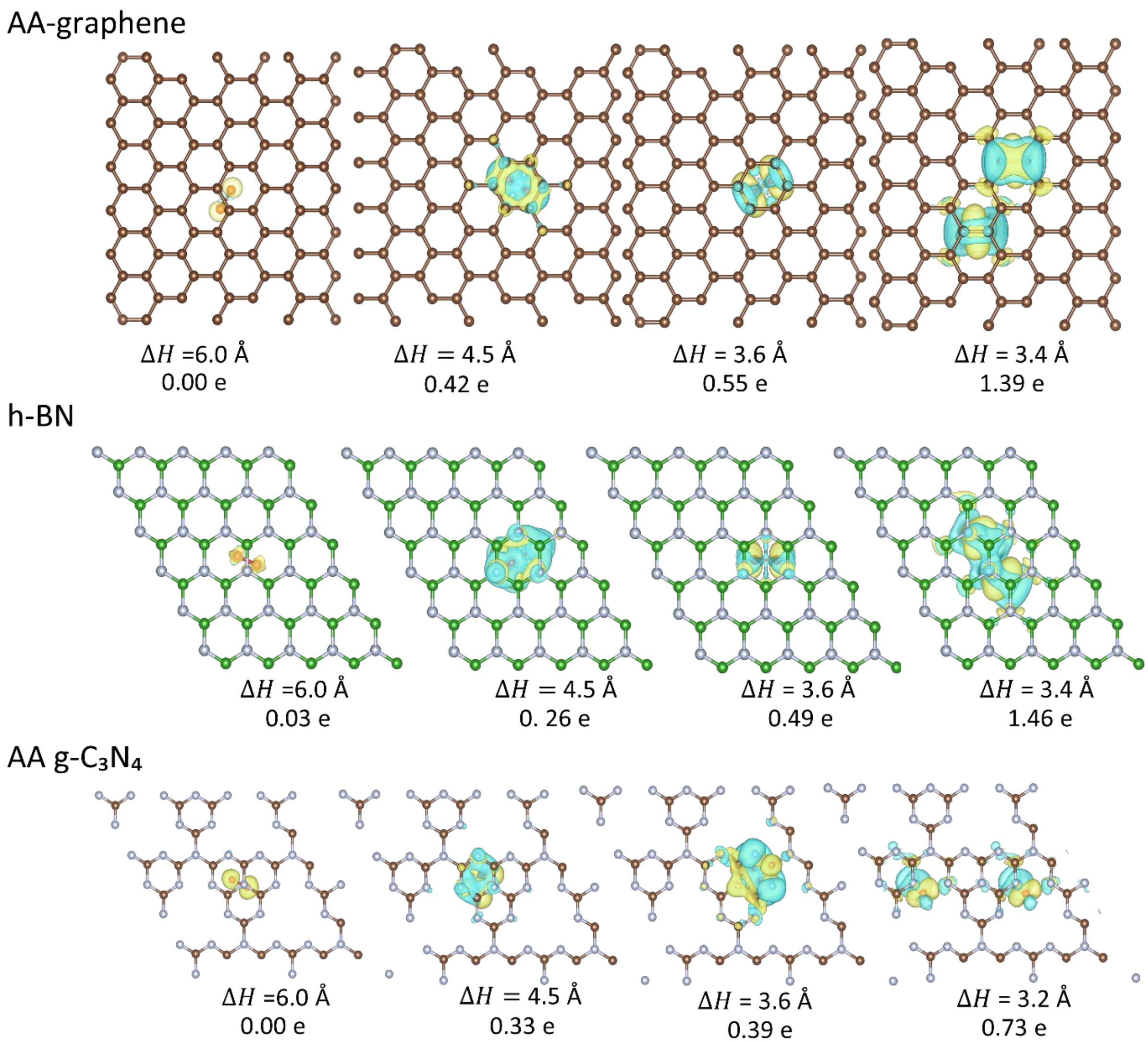

Fig. S2. Top view of Charge transfer, corresponding to FIG.1(d).

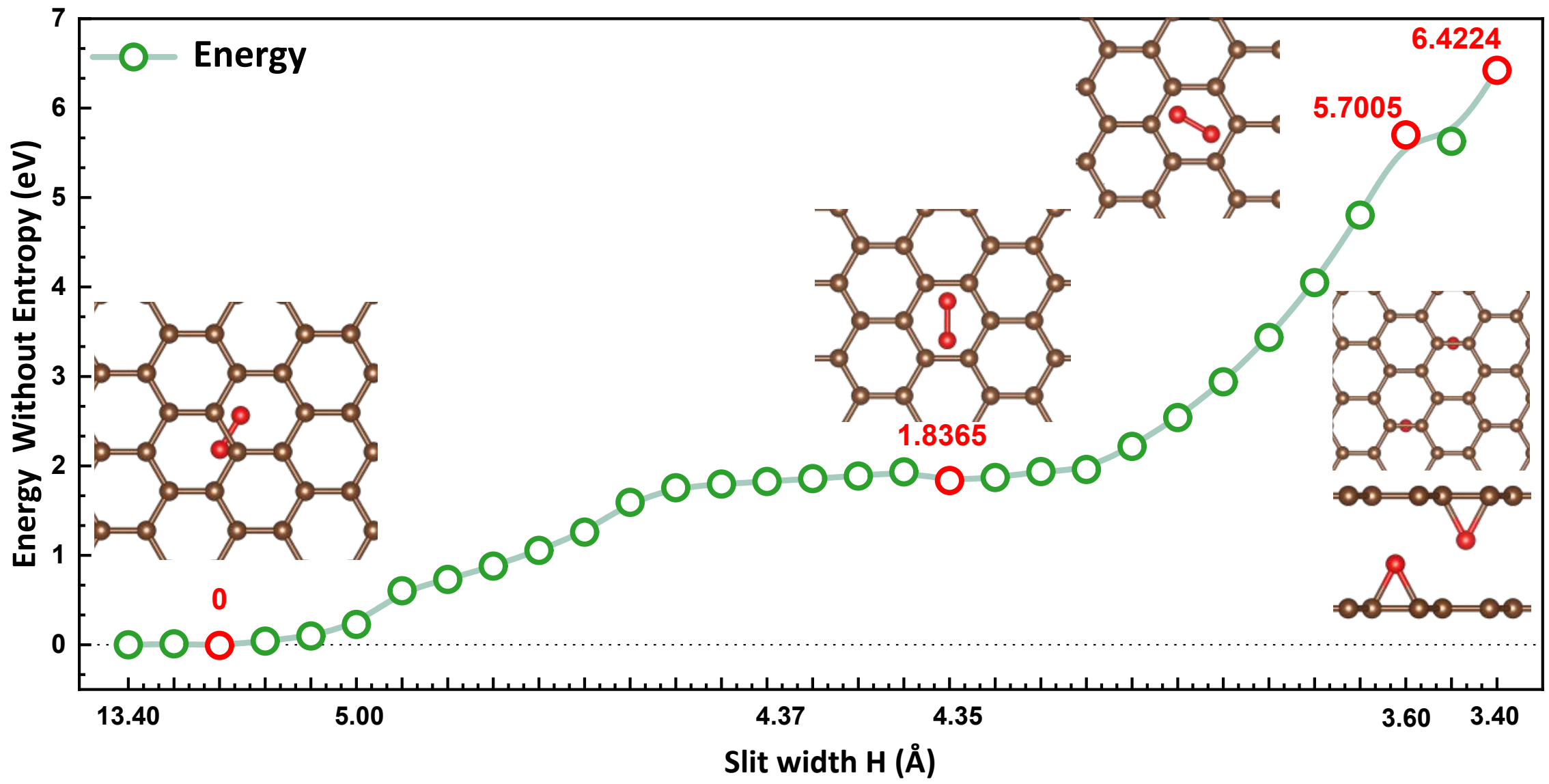

Fig. S3. Energy variation of O_2 under graphene interlayer confinement.

Green circles denote the lowest-energy electronic solutions obtained at each fixed H after full relaxation. Red circles highlight representative regimes: (i) physisorbed triplet at large H (O–O ≈ 1.21 Å, negligible charge transfer, adsorption energy ≈ −0.09 eV), (ii) superoxide-like state at H ≈ 5.7 Å with O–O elongated to 1.27 Å, partial charge transfer, and reduced spin moment, (iii) peroxo-like state at H ≈ 3.6–3.4 Å where spin quenches and O–O bond elongates beyond 1.36 Å, and (iv) dissociated chemisorption near H ≈ 3.4 Å, where each O binds to the graphene walls forming epoxide-like C–O bonds. Insects show optimized structures at selected H values, viewed both parallel and perpendicular to the slit. The monotonic energy rise with decreasing H corresponds to increasing steric/electronic repulsion, while discontinuities and sharp slope changes mark electronic crossovers consistent with the state map in the main text, see FIG. 1.

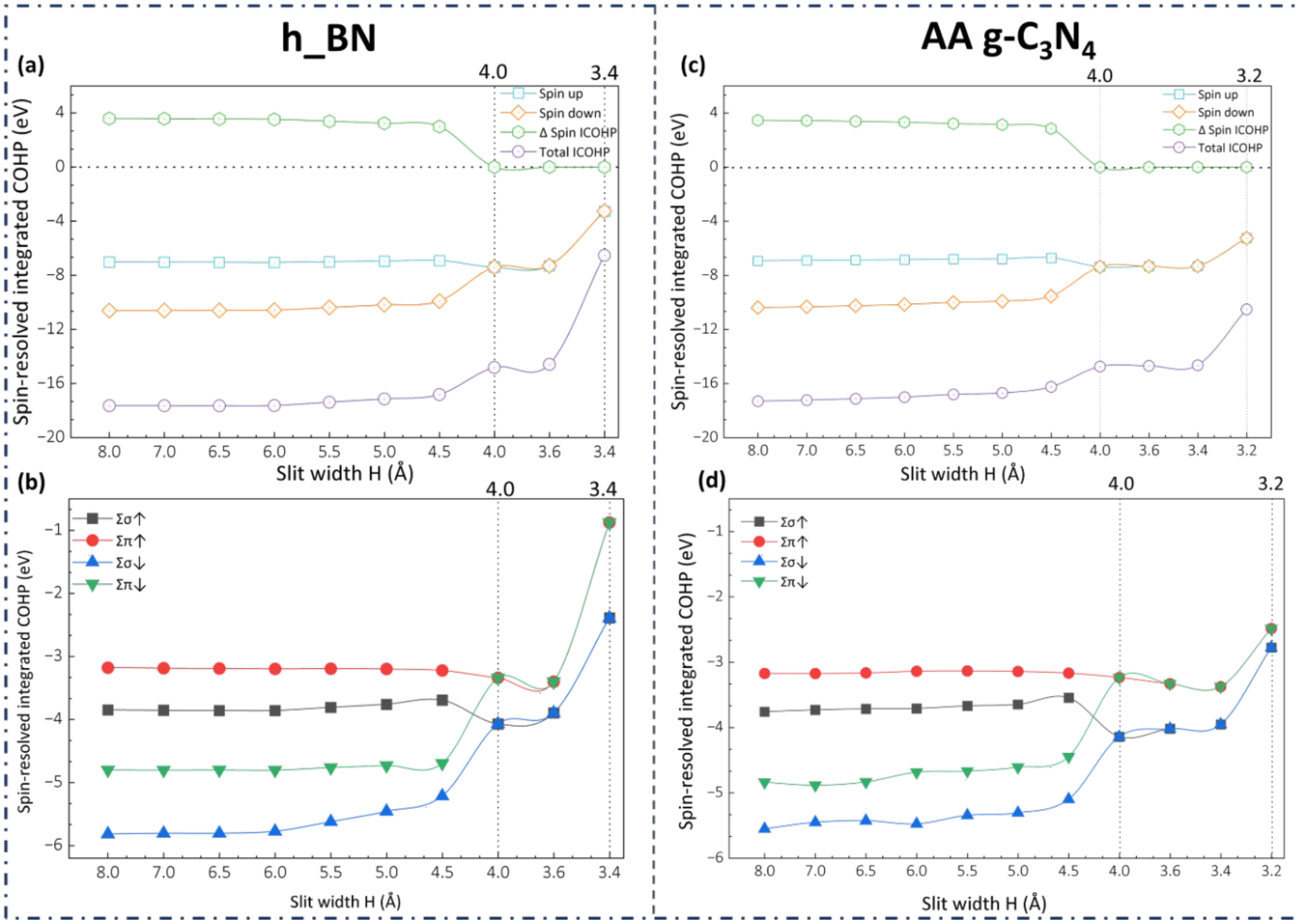

Fig. S4. Integrated COHP decomposition reveals σ/π orbital contributions to O–O bond weakening. (a,c) Spin-resolved ICOHP and ΔICOHP for h-BN and g-C_3N_4, showing spin quenching near $H \approx$ 4.0 Å. (b,d) Integrated bonding contributions from σ and π orbitals. The π channel (especially π↑ and π↓) dominates the bond weakening and spin crossover, while σ contributions remain nearly constant. These results affirm that O_2 activation is π^*-driven and material-independent.

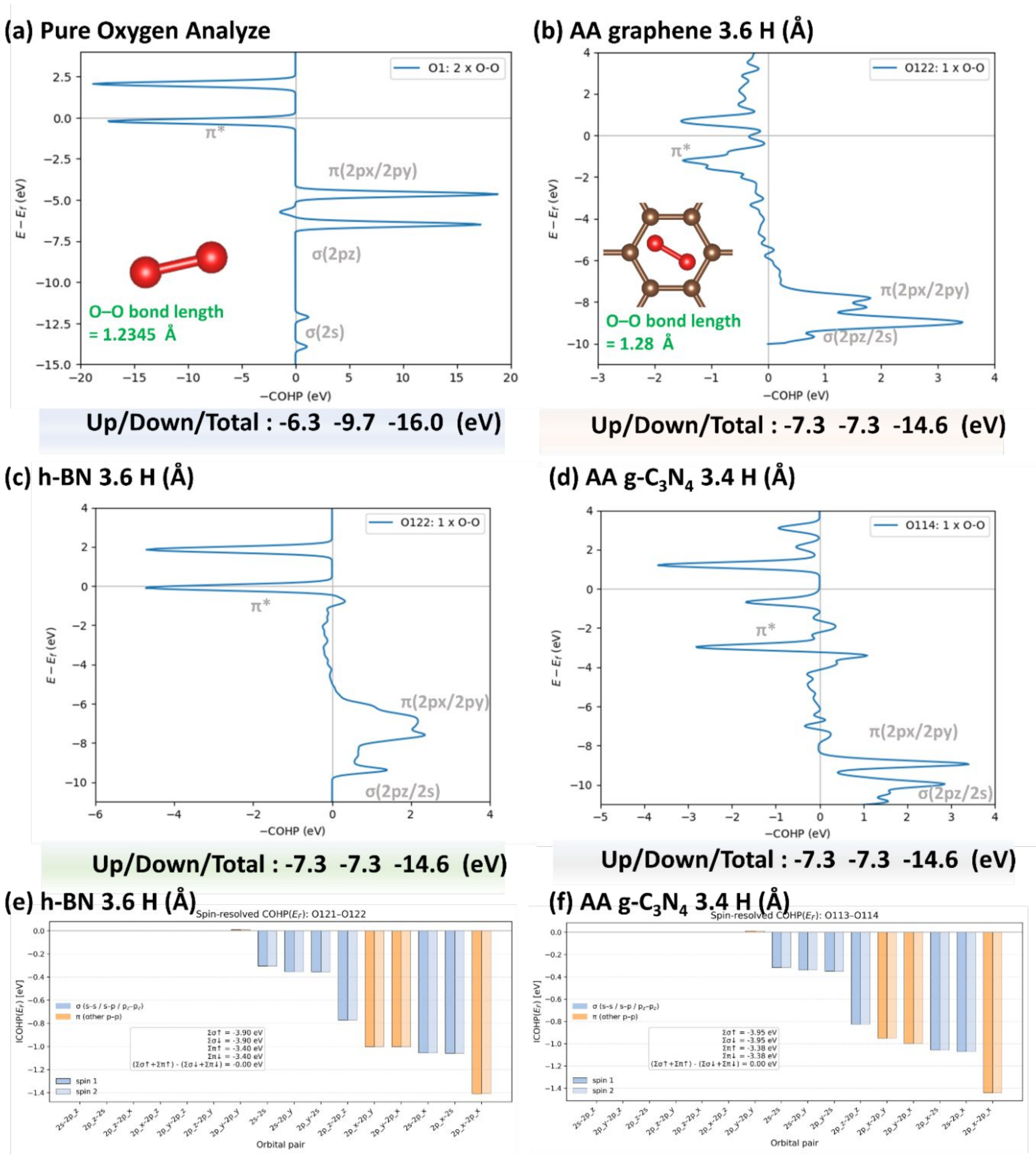

Fig. S5. Energy-resolved −COHP(E) curves for the O–O interaction. (a) For isolated O_2, bonding contributions arise from σ(2s), σ(2pz), and π(2px/2py) channels, while the antibonding π^* lies just above the Fermi level, consistent with a triplet O_2 and an O–O bond length of 1.2345 Å. (b) Under confinement at H = 3.6 Å, the π^* states shift downward toward E_f, becoming partially occupied, which weakens the O–O bond (elongated to 1.28 Å). This redistribution from bonding to antibonding channels illustrates the electronic origin of bond weakening under slit-pore confinement, in line with the observed state map transitions.

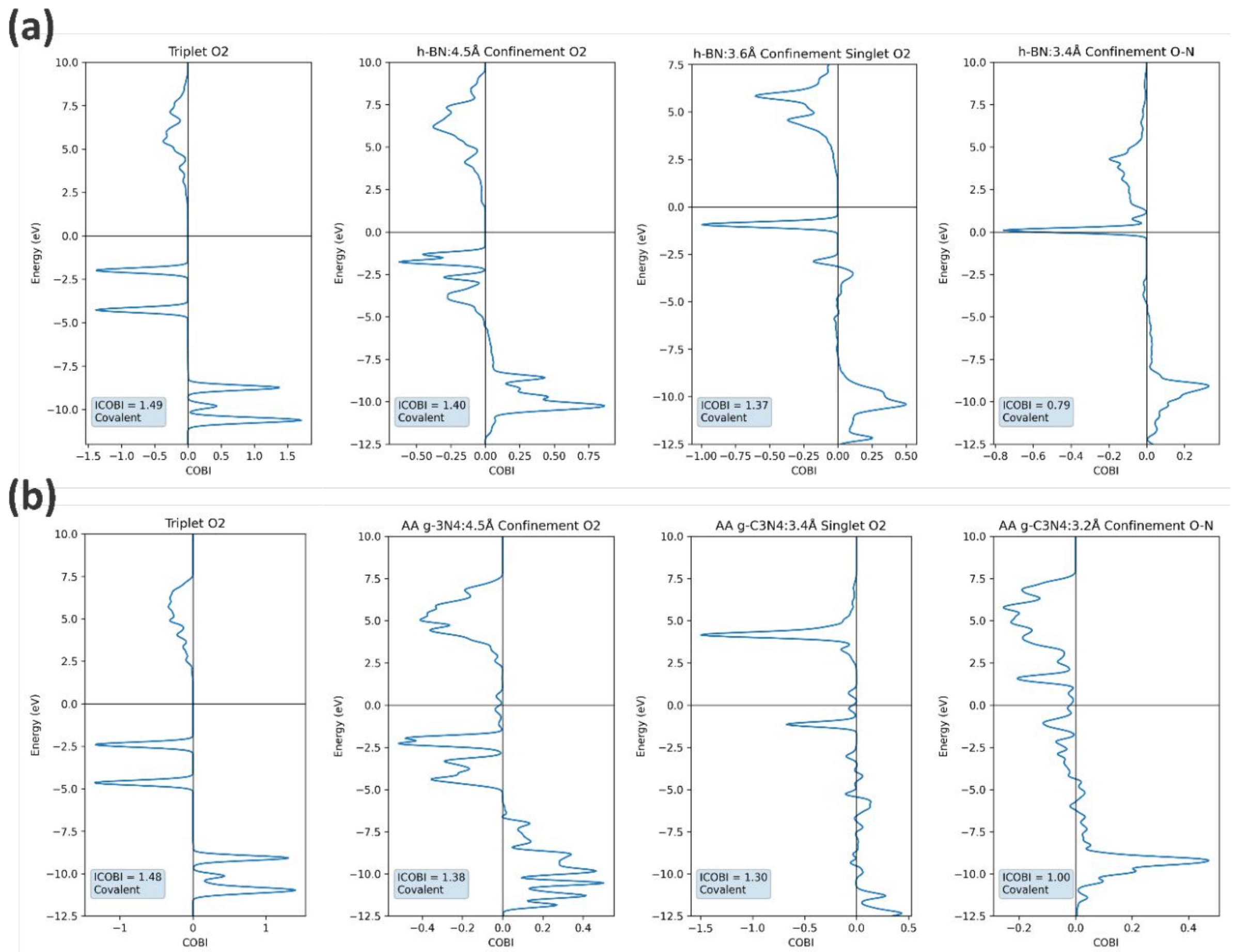

Fig. S6. COBI profiles of the O–O bond under confinement for (a) h-BN, and (b) g-C_3N_4. ICOBI decreases from 1.49 to 1.30 as gap H narrows, indicating spin quenching and bond order reduction toward dissociation.

Energy-resolved Crystal Orbital Bond Index (COBI) plots of the O–O bond in confined environments for (a) h-BN, and (b) g-C_3N_4. Each row compares the free O_2 triplet with sequentially compressed states (H = 6.0, 4.5, 3.6 Å for h-BN; 3.4 Å for g-C_3N_4). ICOBI values systematically decrease from 1.49 (triplet) toward ~1.30 (singlet), reflecting suppression of spin asymmetry and covalent bond weakening. This evolution signals a universal confinement-induced spin quenching and O–O bond-order reduction preceding dissociation. The transition mirrors the textbook triplet-to-singlet conversion mechanism via π^* electron pairing[17], but here uniquely driven by geometric pressure rather than chemical additives. The differing compression thresholds among materials (e.g., graphene: 0.2 Å, h-BN: 0 Å, g-C_3N_4: 0.4 Å) further underscore the role of host stiffness in tuning spin crossover and bond reactivity.

Notes: Although the formal bond order of free triplet O_2 is 2.0, our calculated ICOBI using the LOBSTER projection scheme yields ≈1.48. This deviation is consistent with previous reports using

plane-wave DFT and orbital projection analysis[18], and reflects known limitations from basis set incompleteness, spin contamination, and partial π orbital occupation. Importantly, the ICOBI provides a consistent descriptor for monitoring bond evolution under confinement, even if the absolute value differs from textbook definitions.

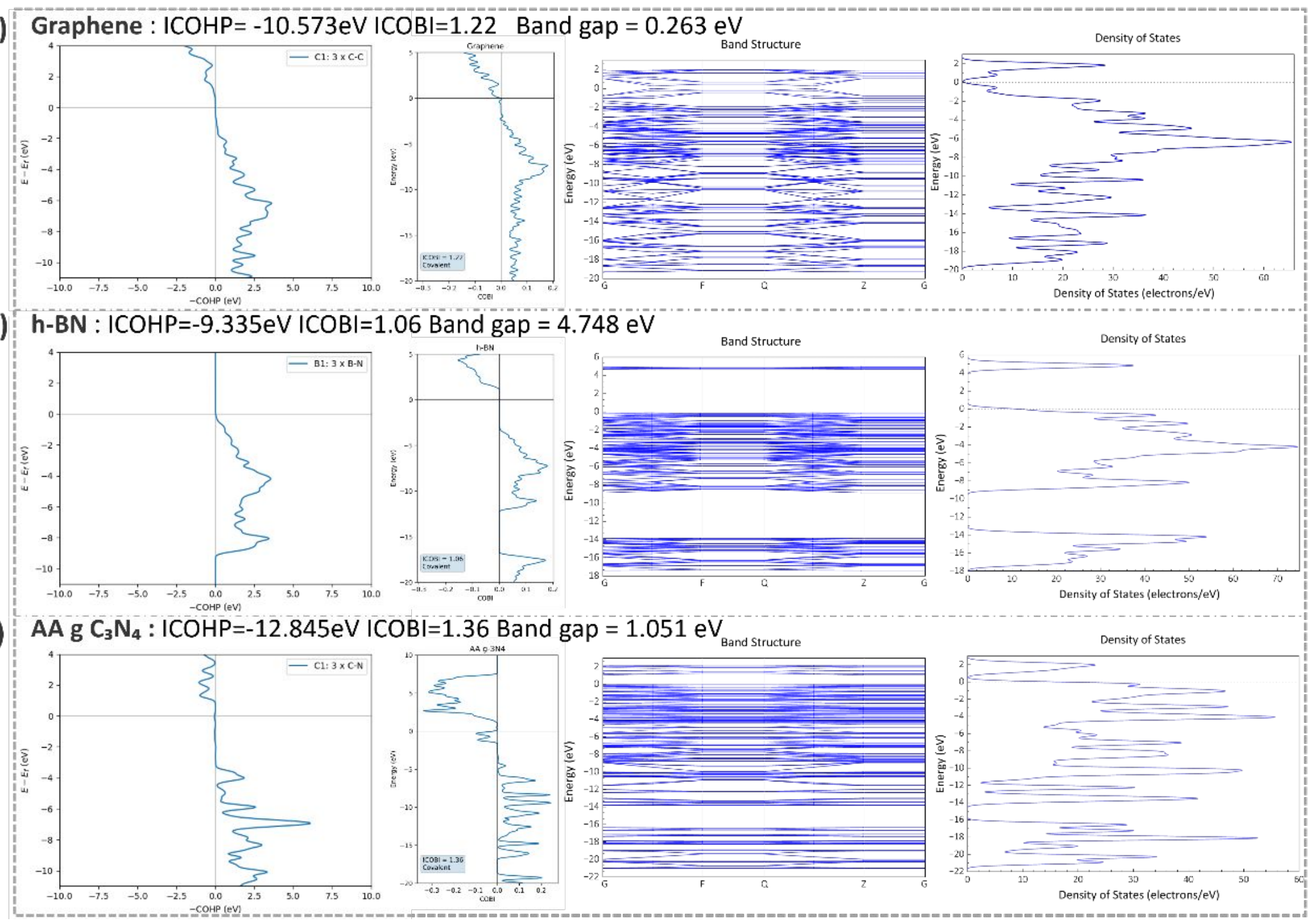

Fig. S7. Electronic and bonding characteristics of pristine confinement walls, corresponding to descriptors listed in Table S4. (a) Graphene, (b) h-BN, and (c) g-C_3N_4: projected COHP (left), energy-resolved ICOBI (middle), and band structure with DOS (right). ICOHP and ICOBI values quantify total bond strength and intralayer covalency, respectively.

A combined analysis of the band gap, intralayer bonding (ICOBI), and total bond strength (ICOHP) of graphene, h-BN, and g-C_3N_4 slit pores can unify their distinct dissociation behaviors. Each of these properties govern how confinement translates into orbital perturbation. In h-BN, O_2 dissociates precisely at the intrinsic interlayer equilibrium distance (approximately 3.4 Å), which is enabled by its low ICOBI (1.06) and weak bonding strength (ICOHP = −9.34 eV). These properties yield the softest structural response among the three materials. This structural pliability enables the O–O bond to be destabilized by physical proximity alone, without the need for additional compression. In contrast, while graphene is electronically favorable for hybridization due to its semimetallic nature and near-zero band gap (0.263 eV)[19], it has moderately strong

intralayer bonding (ICOBI = 1.22 and ICOHP = −10.57 eV). This requires slight over compression (approximately 0.2 Å below its 3.6 Å baseline) to reach the critical orbital interaction regime. Pristine graphene is semimetallic, with the π and π* bands touching at the Dirac K/K′ points in the primitive-cell Brillouin zone. The apparent ~0.26 eV gap-like feature in the finite-supercell band plot is therefore not interpreted as an intrinsic band gap, but as a finite-supercell/k-path effect caused by band folding and by whether the plotted k-point path passes exactly through the folded Dirac point. Similar supercell-folding effects for graphene Dirac points have been discussed previously[20-23].

g-C_3N_4 represents the opposite limit. Despite possessing mid-gap semiconducting character (band gap = 1.05 eV), which in principle supports electronic coupling, its rigid, AA-stacked architecture, as well as its highest ICOBI (1.36) and ICOHP (−12.85 eV), make it the least structurally compliant. This delays O_2 dissociation until approximately 0.4 Å of over compression beyond its 3.6 Å equilibrium. This unified trend highlights a general confinement principle: the onset of reactive orbital mixing is not solely dictated by electronic structure (e.g., band gap), but is also modulated by the wall's mechanical softness, as quantified by ICOBI and ICOHP. This renders h-BN an archetype of deformation-driven activation, while g-C_3N_4 is an example of rigid-state delayed triggering.

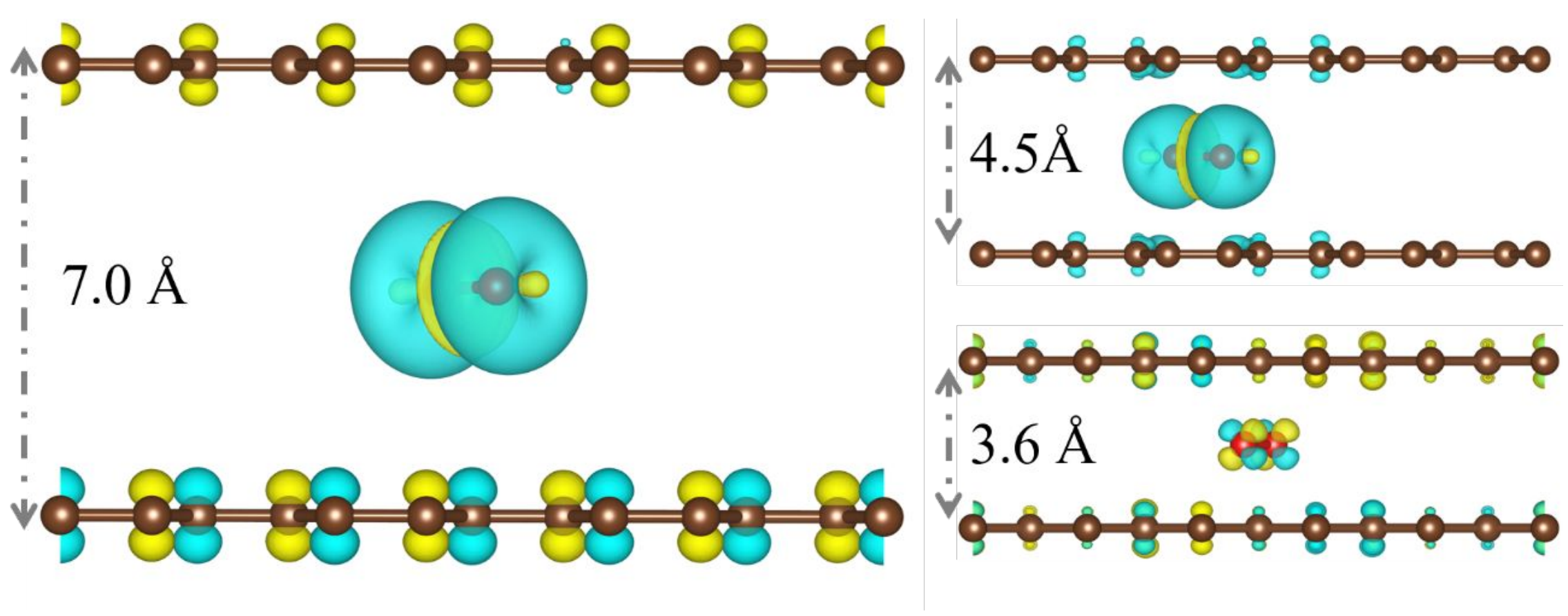

Figure S8. Evolution of the spin-density distribution, $\Delta\rho_s(\mathbf{r}) = \rho_\uparrow(\mathbf{r}) - \rho_\downarrow(\mathbf{r})$, for O_2 confined between graphene walls at $H = 7.0$, 4.5, and 3.6 Å. Green and yellow isosurfaces represent positive spin density ($\rho_\uparrow > \rho_\downarrow$, spin-up) and negative spin density ($\rho_\downarrow > \rho_\uparrow$, spin-down), respectively. Isovalues of $\pm 0.001\, e\, \grave{A}^{-3}$are used for $H = 7.0$and 4.5 Å. For $H = 3.6$Å, the isovalue is reduced to $\pm 4 \times 10^{-6}\, e\, \grave{A}^{-3}$solely to visualize the extremely weak residual spin density; no isosurface would be visible at the common threshold of $0.001\, e\, \grave{A}^{-3}$.

To follow the evolution of the magnetic state along the confinement coordinate, we examined the

real-space spin density, $\Delta\rho_s(\mathbf{r}) = \rho_\uparrow(\mathbf{r}) - \rho_\downarrow(\mathbf{r})$, together with the total-cell and signed O-projected magnetic moments. At $H = 7.0$Å, the spin density is concentrated predominantly around O_2, and the two O atoms carry parallel local moments, indicating a triplet-like state. At $H = 4.5$Å, both the O-centered spin density and the cell magnetic moment are reduced, corresponding to a partially spin-polarized intermediate state. At $H = 3.6$Å, no spin-density isosurface is detectable at the common threshold of $0.001\ e\ \grave{A}^{-3}$, and both the total and O-projected moments approach zero, demonstrating quenching of the O-centered spin polarization under strong confinement. No substantial oppositely signed O-centered moments were found over the investigated coordinate.

3. Supplementary tables

(1). LOBESTER analysis of spin populations and magnetic moments vs *H*

Table S1. Spin populations and magnetic moments of O_2 vs H.

Atom	Orbital	Pure O_2	5.0	4.35	3.6	3.4
	Basis function	Spin up/down	Spin up/down	Spin up/down	Spin up/down	Spin up/down
O_1	2s	0.92/0.91	0.92 /0.94	0.94 /0.94	0.94/0.94	0.95/0.95
	2py	1.00/0.50	0.55 /0.88	0.83 /0.83	0.55/0.55	0.94/0.94
	2pz	0.62/0.63	0.57 /0.99	0.67 /0.67	0.71/0.71	0.59/0.59
	2px	1.00/0.50	0.57 /0.67	0.65 /0.65	0.89/0.89	0.75/0.75
	Total	3.54/2.54	2.61 /3.47	3.09 /3.09	3.08/3.08	3.23/3.23
	M_S	1.00	0.86	0.00	0.00	0.00
O_2	2s	0.92/0.92	0.92 /0.94	0.94 /0.94	0.94/0.94	0.95/0.95
	2py	1.00/0.50	0.55 /0.88	0.83 /0.83	0.55/0.55	0.94/0.94
	2pz	0.54/0.55	0.57 /0.99	0.67 /0.67	0.71/0.71	0.59/0.59
	2px	1.00/0.50	0.57 /0.67	0.65 /0.65	0.89/0.89	0.75/0.75
	Total	3.46/2.46	2.61 /3.47	3.09 /3.09	3.09/3.09	3.23/3.23
	M_S	1.00	0.86	0.00	0.00	0.00

(2). Energy analysis of the system vs H

Table S2. Energies of confined O_2 vs H.

Distance /Å	Total energy /eV	Walls energy /eV	T/S O_2 or 2O energy /eV	Ads T/S O_2 or 2O energy /eV
8.00	-1127.4949	-1117.4807	-9.8670	-0.1177
7.00	-1127.6839	-1117.5948	-9.8630	-0.1926
6.35	-1127.8192	-1117.7080	-9.8620	-0.2147
6.00	-1127.8572	-1117.7829	-9.8610	-0.1778
5.00	-1127.9322	-1118.3386	-9.8550	0.3029
4.70	-1127.7706	-1118.6301	-9.8500	0.7561
4.35	-1127.3958	-1119.0463	-8.6710	1.5470
4.00	-1126.8907	-1119.5703	-8.6680	2.5762
3.80	-1126.1466	-1119.8252	-8.6700	3.5752
3.60	-1124.8688	-1119.9605	-8.6750	4.9883
3.50	-1123.9973	-1119.9521	-8.6820	5.8513
3.40	-1124.3240	-1119.8119	/	/
Pure O_2	-9.90	/	-9.90	/

(3). Mechanical analysis of the system vs H

Table S3. In-plane cell area $\boldsymbol{A}_{\text{cell}}$/ Å² Values for All Wall Materials

Material	a (Å)	b (Å)	γ (°)	A_{cell} / Å²
Graphene	12.7825	12.3000	90°	157.2
h-BN	12.5621	15.0746	120°	163.7
g-C_3N_4	14.2833	14.2833	120°	176.7

Area Calculation for $P_{zz}^{(ex)}(H)$ Normalization

Calculation of A_{cell} (x–y projected area) for pressure normalization

The excess normal pressure $P_{zz}^{(ex)}(H)$ was computed from the adsorption energy slope using:

$$P_{zz}^{(ex)}(H) = -\frac{1}{A_{\text{cell}}} \cdot \frac{dE_{\text{ads}}}{dH}$$

Here, A_{cell} denotes the x–y projected area of the simulation cell, defined from the in-plane lattice vectors as:

$$A_{\text{cell}} = a \cdot b \cdot \sin(\gamma)$$

where $a$and $b$are the lengths of the first two lattice vectors, and $\gamma$is the in-plane angle between them. The calculated values for each material are:

Graphene:

$$a = 12.7825\ \text{Å}, b = 12.3000\ \text{Å}, \gamma = 90°$$

$$A_{\text{graphene}} = 12.7825 \times 12.3000 \times \sin(90°) = 157.2\text{Å}^2$$

h-BN:

$$a = 12.5621\ \text{Å}, b = 15.0746\ \text{Å}, \gamma = 120°$$

$$A_{\text{h-BN}} = 12.5621 \times 15.0746 \times \sin(120°) = 163.7\text{Å}^2$$

g-C_3N_4:

$$a = b = 14.2833\ \text{Å}, \gamma = 120°$$

$$A_{\text{g}-\text{C}_3\text{N}_4} = 14.2833 \times 14.2833 \times \sin(120°) = 176.7\ \text{Å}^2$$

These A values were used consistently to normalize all pressure values reported in the main text and supporting information.

Data for graphene, h-BN, and g-C_3N_4 show sharp pressure buildup as H decreases, fitted to the single exponential form. The best-fit parameters are summarized as follows (see FIG.. 4):

Graphene: $P_\infty = -0.0127$ GPa, $A_1 = 2194.3$ GPa, $L = 0.644$ Å (adjusted $R^2 = 0.996$)

h-BN: $P_\infty = -0.0601$ GPa, $A_1 = 931.25$ GPa, $L = 0.728$ Å (adjusted $R^2 = 0.994$)

g-C_3N_4: $P_\infty = -0.0161$ GPa, $A_1 = 2208.7$ GPa, $L = 0.623$ Å (adjusted $R^2 = 0.998$)

Calculation detail: With the lattice constant 2.46 Å for graphene, $A \approx 157.2$ Å^2. A decrease in H that raises E_{ads}, see Table S2, would yield a positive compressive $P_{zz}^{(ex)}$. At the triplet region of $H = 6.175$ Å, taking the 6.35→6.0 Å interval gives $\Delta E_{ads} = E_{ads}(6.35\ \text{Å}) - E_{ads}(6.0\ \text{Å}) = 0.0369\ eV$ over $\Delta H = -0.35$ Å. Therefore, $dE_{ads}/dH = -0.1054\ eV\text{Å}^{-1}$,and $P_{zz}^{(ex)}$ (6.175 Å) $\approx 0.1074\ GPa$. Near the spin/charge crossover region of $H \approx 4.35$ Å, the triplet-to-anionic/singlet transformation suggests a non-smooth change to both the slope and the $P_{zz}^{(ex)}$. Just above the crossover, from 5.0 Å to 4.7 Å, we have $dE_{ads}/dH \approx -1.5107\ eV\text{Å}^{-1}$, which yields

$P_{zz}^{(ex)}(4.7\ Å^{+}) \approx 1.5397\ GPa$. While for the just below crossover of 4.35 Å to 4.0 Å, we obtain $dE_{ads}/dH \approx -2.9404\ eVÅ^{-1}$ and $P_{zz}^{(ex)}(4.35\ Å^{-}) \approx 2.9968\ GPa$.Such apparent jump is the mechanical correlate of the first-order-like electronic change driven by decreasing H. Processing to the peroxo-like pre-dissociation region, from 3.6 Å to 3.5 Å, $dE_{ads}/dH \approx -8.6300\ eVÅ^{-1}$ and $P_{zz}^{(ex)}(3.55\ Å) \approx 7.2013\ GPa$, which is the largest compressive load before the O–O bond breaking. Qualitatively, the normal load reaches the maximum just before the O–O bond breaking and then redistributes into localized C–O bonds, which would lower the excess normal pressure while tangential stresses become large in the near-wall region, behavior well known from slit-pore pressure-tensor analyses.

(4). Comparative Table: Electronic Indicators and Physical Implications

Table S4. Comparative Summary of Band Structure and Bonding Descriptors for Confinement-Reactive 2D Materials

Material	Band Gap (eV)	AA-Stacked	ICOBI	ICOHP (eV)	Structural Rigidity	Over compression for O_2 Dissociation	Material Property Implication
Graphene	0.263	Yes	1.22	−10.57	Moderate	~0.2 Å (from 3.6 to 3.4 Å)	zero-band-gap semiconductor[19]; π-active; moderately compliant
h-BN	4.748	Yes	1.06	−9.34	Softest	None (3.4 Å intrinsic)	Insulating; weakly covalent; mechanically soft
g-C_3N_4 (AA)	1.051	Yes	1.36	−12.85	Rigidest	~0.4 Å (from 3.6 to 3.2 Å)	Semiconducting; strongly covalent; mechanically rigid

Table S4 compares host descriptors: band gap, ICOBI, and ICOHP. g-C_3N_4 shows the strongest intralayer covalency (ICOBI = 1.36) and deepest bond energy (ICOHP = -12.84 eV), making it most rigid and requiring the greatest compression for O_2 dissociation. h-BN, with lower bond metrics (ICOBI = 1.06, ICOHP = -9.34 eV), is structurally softer, consistent with its dissociation point coinciding with equilibrium.

(5). Materials-independence of confinement-induced O_2 activation.

Table S5. Regime-resolved signatures of material-independent O_2 activation under Å-scale confinement

Host Materials	Rigime	Gap (Å)	M_S (μ_B)	ℓ_{O-O} (Å)	Δq (e^-)	$P_{zz}^{(ex)}(H)$
h-BN	I/II	8.0-4.5	2	1.233-1.244	0-0.257	0-1.651
	III	4.0-3.5	0	1.273-1.277	0.436-0.489	2.523-7.200
	IV	Below 3.4	0	2.513	1.461	>7.200
g-C_3N_4	I/II	8.0-4.5	1.995-1.885	1.238-1.255	0-0.334	0-2.343
	III	4.0-3.4	0	1.274-1.281	0.380-0.385	4.794-8.084
	IV	Below 3.2	0	7.143	0.73	>8.08

This consistency reinforces that the O_2 transformations are driven by confinement physics rather than any specific chemistry of the walls.

(6). Effect of spin–orbit coupling on the confinement-dependent spin state.

To assess the influence of spin–orbit coupling (SOC), static noncollinear calculations were performed for representative graphene-confined O_2 structures using LSORBIT = .TRUE. and LNONCOLLINEAR = .TRUE., with the initial magnetization axis oriented along z. No constraint was imposed on the final total magnetization.

Table S6. Total and O-projected magnetic moments obtained from noncollinear spin–orbit-coupled calculations for O_2 confined between graphene walls. All magnetic moments are reported in μ_B.

H(Å)	M_x	M_y	M_z	$\|\mathbf{M}\|$	$M_\perp$	$\|\mathbf{m}_{O1}\|$	$\|\mathbf{m}_{O2}\|$	**Electronic-state assignment**
3.4	0.000	0.000	0.000	0.000	0.000	<0.001	<0.001	Spin-quenched
4.0	0.000	0.000	0.000	0.000	0.000	<0.001	<0.001	Spin-quenched
4.5	0.000	−0.001	1.995	1.995	0.001	0.660	0.660	Triplet-like
6.0	−0.001	0.000	2.000	2.000	0.001	0.785	0.787	Triplet-like

Notes: The total magnetization vector is defined as $\boldsymbol{M} = (M_x, M_y, M_z)$*, with* $|\boldsymbol{M}| = (M_x^2 + M_y^2 +$

$M_z^2)^{1/2}$ *and* $M_\perp = (M_x^2 + M_y^2)^{1/2}$. *The initial magnetization axis was oriented along* z. *The O-projected magnetic moments were integrated within the PAW projection spheres and therefore do not necessarily sum to the full-cell magnetization. At* $H = 4.5$ *and 6.0 Å, the transverse magnetizations correspond to canting angles of approximately* 0.028° *and* 0.026°*, respectively. Canting angles are not reported for the spin-quenched structures because their total magnetic moments are numerically negligible.*

Table S6 summarizes the total magnetization vectors and O-projected magnetic moments obtained from the noncollinear SOC calculations. Explicit inclusion of SOC preserves the confinement-dependent spin-state evolution. At $H = 3.4$ and 4.0 Å, the magnitudes of the total magnetization vectors are only 1.03×10^{-5} and 3.24×10^{-5} μ_B, respectively. The projected magnetic moments on both O atoms are also below 0.001 μ_B. Thus, the compressed structures remain spin-quenched and show no appreciable residual O-centered local spin polarization.

At $H = 4.5$ and 6.0 Å, the magnitudes of the total magnetization vectors are 1.9947 and 1.9998 μ_B, respectively, consistent with triplet-like O_2. The two O atoms carry parallel z-projected moments of 0.660 and 0.660 μ_B at 4.5 Å and 0.785 and 0.787 μ_B at 6.0 Å. The difference between the full-cell magnetization and the sum of the atom-projected moments arises from magnetization distributed over the graphene wall and the interstitial region outside the PAW projection spheres. The transverse magnetizations at 4.5 and 6.0 Å are only 9.79×10^{-4} and 8.96×10^{-4} μ_B, corresponding to canting angles of approximately 0.028° and 0.026°, respectively. These very small noncollinear components are consistent with the weak intrinsic SOC expected for a light-element C/O system. The spin-state crossover therefore remains between 4.0 and 4.5 Å when SOC is explicitly included.

(7). Signed magnetic moments, spin alignment, and spin-state assignments.

Table S7. Total-cell and signed O-projected magnetic moments of O_2 confined between graphene, h-BN, and g-C_3N_4 walls. All magnetic moments are reported in μ_B.

Wall	H(Å)	M_{cell}	m_{O1}	m_{O2}	$\lvert m_{\text{O1}}\rvert + \lvert m_{\text{O2}}\rvert$	Alignment	Assignment
Graphene	3.40	0.000	0.000	0.000	0.000	Quenched	Spin-quenched
Graphene	3.60	0.000	0.000	0.000	0.000	Quenched	Spin-quenched
Graphene	3.80	0.000	0.000	0.000	0.000	Quenched	Spin-quenched
Graphene	4.00	0.000	0.000	0.000	0.000	Quenched	Spin-quenched
Graphene	4.35	0.000	0.000	0.000	0.000	Quenched	Spin-quenched

Graphene	4.50	−1.382	−0.569	−0.568	1.137	Parallel	Partially spin-polarized/intermediate
Graphene	4.70	−1.546	−0.656	−0.662	1.318	Parallel	Partially spin-polarized/intermediate
Graphene	5.00	−1.586	−0.703	−0.707	1.410	Parallel	Partially spin-polarized/intermediate
Graphene	7.00	−1.782	−0.768	−0.768	1.536	Parallel	Triplet-like
h-BN	3.40	0.000	0.000	0.000	0.000	Quenched	Spin-quenched
h-BN	3.60	0.000	0.000	0.000	0.000	Quenched	Spin-quenched
h-BN	4.00	0.000	0.000	0.000	0.000	Quenched	Spin-quenched
h-BN	4.50	2.000	0.727	0.716	1.443	Parallel	Triplet-like
h-BN	5.00	2.000	0.760	0.765	1.525	Parallel	Triplet-like
h-BN	5.50	2.000	0.785	0.784	1.569	Parallel	Triplet-like
h-BN	6.00	2.000	0.802	0.803	1.605	Parallel	Triplet-like
h-BN	6.50	2.000	0.806	0.807	1.613	Parallel	Triplet-like
h-BN	7.00	2.000	0.808	0.809	1.617	Parallel	Triplet-like
h-BN	8.00	2.000	0.810	0.810	1.620	Parallel	Triplet-like
g-C_3N_4	3.20	0.000	0.000	0.000	0.000	Quenched	Spin-quenched
g-C_3N_4	3.40	0.000	0.000	0.000	0.000	Quenched	Spin-quenched
g-C_3N_4	3.60	0.000	0.000	0.000	0.000	Quenched	Spin-quenched
g-C_3N_4	4.00	0.000	0.000	0.000	0.000	Quenched	Spin-quenched
g-C_3N_4	4.50	2.000	0.704	0.682	1.386	Parallel	Triplet-like
g-C_3N_4	5.00	1.991	0.749	0.744	1.493	Parallel	Triplet-like
g-C_3N_4	5.50	1.929	0.748	0.764	1.512	Parallel	Triplet-like
g-C_3N_4	6.00	1.933	0.767	0.781	1.548	Parallel	Triplet-like
g-C_3N_4	6.50	1.951	0.780	0.793	1.573	Parallel	Triplet-like
g-C_3N_4	7.00	1.966	0.789	0.800	1.589	Parallel	Triplet-like
g-C_3N_4	8.00	1.981	0.798	0.804	1.602	Parallel	Triplet-like

Notes: The signs of the magnetic moments indicate the arbitrary global orientation of the collinear spin axis. The relative signs of m_{O1} and m_{O2}, rather than their common absolute sign, distinguish parallel from antiparallel O-centered spin alignment. The reported atomic moments are PAW-sphere-projected quantities.

Because an explicit approximate-spin-projection correction was not applied, possible residual spin mixing was not quantitatively evaluated in the present work[24, 25]. The terms 'triplet-like,' 'partially spin-polarized,' and 'spin-quenched' therefore describe the calculated magnetic-moment and spin-density patterns rather than rigorously spin-pure eigenstates. More rigorous spin-projected or multireference treatments of periodic systems could be addressed in future work.

(8). HSE06-D3(BJ) validation of confinement-dependent O_2 structures and spin states.

Table S8. Selected structural and magnetic properties of confined O_2 obtained from constrained HSE06-D3(BJ) geometry optimizations.

Wall material	Slit width, H (Å)	O–O distance (Å)	|M| (μB)
Graphene	3.4	2.4124	0.0000
Graphene	4.0	1.2507	0.0000
Graphene	4.5	1.2157	2.0000
Graphene	6.0	1.2102	2.0000
h-BN	3.4	2.4955	0.0000
h-BN	4.0	1.2400	0.0000
h-BN	4.5	1.2123	2.0000
h-BN	6.0	1.2100	2.0000
g-C_3N_4	3.4	1.2486	0.0000
g-C_3N_4	4.0	1.2521	0.0000
g-C_3N_4	4.5	1.2184	2.0000
g-C_3N_4	6.0	1.2107	2.0000

Notes: *The HSE06-D3(BJ) calculations were initiated from the corresponding PBE-D3(BJ)-optimized structures and performed using Γ-point sampling. The wall atoms and slit width were kept fixed, while only the atomic coordinates of O_2 were relaxed. The magnitude of the total magnetic moment is reported.*

(9). Dependence of the relaxed O_2 configuration on initial orientation and adsorption site.

To assess the dependence on the initial O_2 configuration, additional geometry optimizations were performed using multiple molecular orientations and laterally distinct positions(see Table S9). The initial orientations included configurations parallel to the walls, tilted by approximately 30° and 60°, and, where applicable, perpendicular to the walls. The sampled lateral positions included bridge, hollow, and top sites for graphene; C–N-bridge, N-rich, pore-center, and reactive-N-neighbor environments for g-C_3N_4; and B-top, N-top, B–N-bridge, and hexagon-center positions for h-BN. Each candidate was relaxed independently.

Table S9. Lowest-energy relaxed O_2 structures identified among the tested initial orientations and lateral positions. The final angle, θ_f, is defined as the acute angle between the relaxed O–O axis and the wall plane. The alternative structure is the lowest-energy configuration obtained from a different initial orientation, and $\Delta E_{\mathrm{alt}} = E_{\mathrm{alt}} - E_{\mathrm{min}}$.

Wall material	H (Å)	Starting orientation/site of selected minimum	Final θ_f (°)	Lowest alternative orientation/site	Alternative θ_f (°)	ΔE_{alt} (eV)
Graphene	3.4	Parallel / bridge	0.00	Tilted 30° / top	0.00	0.029
Graphene	4.5	Tilted 60° / bridge	0.04	Parallel / bridge	0.08	0.003
Graphene	6.0	Parallel / hollow	0.00	Tilted 30° / hollow	0.59	<0.001
g-C_3N_4	3.4	Tilted 30° / reactive-N-neighbor	0.13	Parallel / reactive-N-neighbor	0.08	0.272
g-C_3N_4	4.5	Perpendicular / C–N bridge	0.10	Tilted 30° / pore center	0.01	<0.001
g-C_3N_4	6.0	Tilted 30° / N-rich site	0.19	Tilted 60° / N-rich site	0.07	<0.001
h-BN	3.4	Parallel / B–N bridge	0.00	Tilted 30° / B–N bridge	0.01	1.047
h-BN	4.5	Tilted 60° / N-top	1.65	Tilted 30° / N-top	0.26	0.017
h-BN	6.0	Tilted 60° / hexagon center	0.02	Tilted 30° / hexagon center	0.69	<0.001

Notes: The listed orientation and lateral position describe the starting geometry before structural relaxation. For each wall material and slit width, the selected structure is the lowest-energy

relaxed configuration identified among the tested initial geometries. A final angle of 0° corresponds to an O–O axis parallel to the confining walls, whereas 90° corresponds to a perpendicular configuration. Values of ΔE_{alt} below 0.001 eV indicate nearly degenerate relaxed structures.

Taken together, these results define a clear confinement-driven progression: spin quenching → charge redistribution; bond softening; O_2 activation; chemisorption. All trends are captured by consistent variations in spin moment M_S (μ_B), ℓ_{O-O}, Δq, *ICOHP*, and *ICOBI*. This underscores a unified physical picture where Å-level confinement alone can deterministically trigger spin crossover and reactivity, independent of host chemistry.

4. Computational files

A complete set of computational data and analysis codes are provided for the case of H=3.6 Å, "Graphene-H3.6.zip".

REFFERANCE

(1) Blöchl, P. E. Projector augmented-wave method. *Phys. Rev. B* **1994**, 50, 17953.
(2) Grimme, S.; Antony, J.; Ehrlich, S.; Krieg, H. A consistent and accurate ab initio parametrization of density functional dispersion correction (DFT-D) for the 94 elements H-Pu. *Journal of Chemical Physics* **2010**, 132, 154104.
(3) Grimme, S.; Ehrlich, S.; Goerigk, L. Effect of the damping function in dispersion corrected density functional theory. *J Comput Chem* **2011**, 32, 1456-1465.
(4) Matta, C. F.; Bader, R. F. W. Atoms-in-molecules study of the genetically encoded amino acids. II. Computational study of molecular geometries. *Proteins-Structure Function and Genetics* **2002**, 48, 519-538.
(5) Henkelman, G.; Arnaldsson, A.; Jónsson, H. A fast and robust algorithm for Bader decomposition of charge density. *Comput. Mater. Sci.* **2006**, 36, 354-360.
(6) Wang, V.; Xu, N.; Liu, J. C.; Tang, G.; Geng, W. T. VASPKIT: A user-friendly interface facilitating high-throughput computing and analysis using VASP code. *Comput. Phys. Commun.* **2021**, 267, 108033.
(7) George, J.; Petretto, G.; Naik, A.; Esters, M.; Jackson, A. J.; Nelson, R.; Dronskowski, R.; Rignanese, G. M.; Hautier, G. Automated Bonding Analysis with Crystal Orbital Hamilton Populations. *ChemPlusChem* **2022**, 87, e202200123.
(8) Dronskowski, R.; Bloechl, P. E. Crystal orbital Hamilton populations (COHP): energy-resolved visualization of chemical bonding in solids based on density-functional calculations. *J. Phys. Chem.* **1993**, 97, 8617-8624.
(9) Yang, G.; Li, L.; Lee, W. B.; Ng, M. C. Structure of graphene and its disorders: a review. *Sci Technol Adv Mater* **2018**, 19, 613-648.
(10) Choi, J. I.; Kim, H. S.; Sohn, Y.-J.; Yim, S.-D.; Alamgir, F. M.; Jang, S. S. Density Functional Theory Study of Oxygen Reduction on Graphene and Platinum Surfaces of Pt–Graphene Hybrids. *ACS Applied Nano Materials* **2021**, 4, 1067-1075.
(11) Mehmood, F.; Pachter, R.; Lu, W.; Boeckl, J. J. Adsorption and Diffusion of Oxygen on Single-Layer Graphene with Topological Defects. *J. Phys. Chem. C* **2013**, 117, 10366-10374.
(12) Han, C.; Chen, Z. Adsorption properties of O2 on the unequal amounts of binary co-doped graphene by B/N and P/N: A density functional theory study. *Applied Surface Science* **2019**, 471, 445-454.
(13) Krukau, A. V.; Vydrov, O. A.; Izmaylov, A. F.; Scuseria, G. E. Influence of the exchange screening parameter on the performance of screened hybrid functionals. *J. Chem. Phys.* **2006**, 125.
(14) Heyd, J.; Scuseria, G. E.; Ernzerhof, M. Hybrid functionals based on a screened Coulomb potential. *J. Chem. Phys.* **2003**, 118, 8207-8215.
(15) Paier, J.; Marsman, M.; Hummer, K.; Kresse, G.; Gerber, I. C.; Ángyán, J. G. Screened hybrid density functionals applied to solids. *J. Chem. Phys.* **2006**, 124.
(16) Dai, J. Y.; Yuan, J. M. Adsorption of molecular oxygen on doped graphene: Atomic, electronic, and magnetic properties. *Phys. Rev. B* **2010**, 81, 165414.
(17) Steudel, R. De Gruyter, 2020.
(18) Maintz, S.; Deringer, V. L.; Tchougréeff, A. L.; Dronskowski, R. LOBSTER: A tool to extract chemical bonding from plane-wave based DFT. *J Comput Chem* **2016**, 37, 1030-1035.

(19) Wang, J. G.; Ma, F. C.; Sun, M. T. Graphene, hexagonal boron nitride, and their heterostructures: properties and applications. *RSC Adv.* **2017**, 7, 16801-16822.
(20) Castro Neto, A. H.; Guinea, F.; Peres, N. M.; Novoselov, K. S.; Geim, A. K. The electronic properties of graphene. *Rev. Mod. Phys.* **2009**, 81, 109-162.
(21) Ghorbani-Asl, M.; Juarez-Mosqueda, R.; Kuc, A.; Heine, T. Efficient quantum simulations of metals within the Γ-point approximation: Application to carbon and inorganic 1D and 2D materials. *J. Chem. Theory Comput.* **2012**, 8, 2888-2895.
(22) Zhou, Y.-C.; Zhang, H.-L.; Deng, W.-Q. A 3 N rule for the electronic properties of doped graphene. *Nanotechnology* **2013**, 24, 225705.
(23) Lanzara, A. Graphene gets a good gap. *Physics* **2015**, 8, 91.
(24) Tada, K.; Yamanaka, S.; Kawakami, T.; Kitagawa, Y.; Okumura, M.; Yamaguchi, K.; Tanaka, S. Estimation of spin contamination errors in DFT/plane-wave calculations of solid materials using approximate spin projection scheme. *Chemical Physics Letters* **2021**, 765, 138291.
(25) Kitagawa, Y.; Saito, T.; Ito, M.; Shoji, M.; Koizumi, K.; Yamanaka, S.; Kawakami, T.; Okumura, M.; Yamaguchi, K. Approximately spin-projected geometry optimization method and its application to di-chromium systems. *Chemical Physics Letters* **2007**, 442, 445-450.
